# Transcriptome analysis reveals a composite molecular map linked to unique seed oil profile of *Neocinnamomum caudatum* (Nees) Merr

**DOI:** 10.1186/s12870-018-1525-9

**Published:** 2018-11-26

**Authors:** Yi Gan, Yu Song, Yadong Chen, Hongbo Liu, Dongdong Yang, Qianyu Xu, Zhifu Zheng

**Affiliations:** 1 School of Agriculture and Food Sciences, Zhejiang A & F University, Zhejiang, 311300 Hangzhou China; 2 Center for Integrative Conservation, Xishuangbanna Tropical Botanical Garden, Chinese Academy of Sciences, Mengla, 666303 Yunnan China

**Keywords:** *Neocinnamomum caudatum* (Nees) Merr., Lauraceae, Linoleic acid, Stearic acid, Transcriptome sequencing, Acyl-ACP thioesterase B, Heterologous expression

## Abstract

**Background:**

*Neocinnamomum caudatum* (Nees) Merr.*,* a biodiesel tree species in the subtropical areas of South China, India and Burma, is distinctive from other species in Lauraceae family and its seed oil is rich in linoleic acid (18:2) and stearic acid (18:0). However, there is little genetic information about this species so far. In this study, a transcriptomic analysis on developing seeds of *N. caudatum* was conducted in an attempt to discern the molecular mechanisms involving the control of the fatty acid (FA) and triacylglycerol (TAG) biosynthesis.

**Results:**

Transcriptome analysis revealed 239,703 unigenes with an average length of 436 bp and 137 putative biomarkers that are related to FA formation and TAG biosynthesis. The expression patterns of genes encoding β-ketoacyl-acyl carrier protein synthase I (KASI), β- ketoacyl-acyl carrier protein synthase II (KASII), stearoyl-ACP desaturase (SAD), fatty acid desaturase 2 (FAD2), fatty acid desaturase 8 (FAD8) and acyl-ACP thioesterase A/B (FATA/B) were further validated by qRT-PCR. These genes displayed a very similar expression pattern in two distinct assays. Moreover, sequence analysis of different FATBs from diverse plant species revealed that NcFATB is structurally different from its counterpart in other species in producing medium-chain saturated FAs. Concertedly, heterologous expression of *NcFATB* in *E. coli* BL21 (DE3) strain showed that this corresponding expressed protein, NcFATB, prefers long-chain saturated fatty acyl-ACP over medium-chain fatty acyl-ACP as substrate.

**Conclusions:**

Transcriptome analysis of developing *N. caudatum* seeds revealed a composite molecular map linked to the FA formation and oil biosynthesis in this biodiesel tree species. The substrate preference of NcFATB for long-chain saturated FAs is likely to contribute to its unique seed oil profile rich in stearic acid. Our findings demonstrate that in the tree species of Lauraceae family, the FATB enzymes producing long-chain FAs are structurally distinct from those producing medium-chain FAs, thereby suggesting that the *FATB* genes may serve as a biomarker for the classification of tree species of Lauraceae family.

**Electronic supplementary material:**

The online version of this article (10.1186/s12870-018-1525-9) contains supplementary material, which is available to authorized users.

## Background

*Neocinnamomum caudatum* (Nees) Merr.*,* a widely distributed species in the subtropical areas of South China, India and Burma, was assigned to the genus *Neocinnamomum* in the family Lauraceae [1, 2]. Being one of the most enigmatic species of the Lauraceae family, *N. caudatum* shares many morphological similarities with species of the genus *Cinnamomum*. However, the phylogenetic analysis based on chloroplast genome shows that the genus *Neocinnamomum* is monophyletic and evolutionally far away from *Cinnamomum* [3, 4]. Genus *Neocinnamomum* comprises only six species endemic to tropical Asia and shares a close relationship with the genus *Caryodaphnopsis* [3, 5]. In China, *N. caudatum* is also known as “Baigui” whose bark and leaves have long been used as a traditional Chinese medicine [2]. In addition, mature seeds of *N. caudatum* contain up to 57.4 % of the storage lipid TAG on a dry weight basis [6]. In a sharp contrast to many well-documented Lauraceae species that produce predominantly medium-chain fatty acids (MCFA) in their seeds, such as decanoic acid (8:0), capric acid (10:0) and lauric acid (12:0),seeds of *N. caudatum* contain exclusively long-chain fatty acids(LCFA). Palmitic acid (16:0), stearic acid (18:0), oleic acid (18:1), linoleic acid (18:2) and linolenic acid (18:3), respectively account for 11.3, 21.2, 15.8, 35 and 13.1% of the total FAs (expressed as a mole percent) [6, 7]. Notably, such a high proportion of 18:0 is rare in the family Lauraceae and even in the subclass Magnoliidae, implying that the molecular mechanisms governing the FA formation and triacylglycerol biosynthesis in seeds of *N. caudatum* are very likely to be different from those in other well-documented species in the family Lauraceae. Recently, *N. caudatum* has received much attention for its significant seed oil content, distinctive FA profile and abundant fruit yield. It has been recommended as a potential source of biodiesel in China [8]. Surprisingly, however, little is known about its genomic information so far.

In higher plants, the biosynthesis of FA initially takes place in the plastids, starting with pyruvate generated from glycolysis. In the plastids, pyruvate is oxidized to acetyl-CoA, which is then carboxylated by acetyl-CoA carboxylase (ACC) generating malonyl-CoA, the building block of FA synthesis [9]. FAs assembly occurs on acyl carrier protein (ACP) via a cycle of 4 reactions allowing the elongation of the acyl chain by two carbons each cycle. After seven cycles, the saturated 16:0-ACP can either be hydrolyzed by an acyl-ACP thioesterase (FAT) or further elongated by a β-ketoacyl-acyl carrier protein synthase (KASII) to 18:0-ACP. The latter then undergoes two fates: direct hydrolysis by a FAT enzyme or desaturation by SAD to generate 18:1-ACP which is then subjected to further hydrolysis. The free FAs formed from Acyl-ACP are then transported to the cytosol for further desaturation or elongation [10, 11]. It is generally accepted that the FAT enzymes are one of the key determinants of the FA chain length [12]. Based on the substrate preference, there are two types of FATs in plants (FATA and FATB). FATA prefers unsaturated acyl-ACP (such as 16:1-ACP and 18:1-ACP ), while FATB prefers saturated acyl-ACP (such as 16:0-ACP and 18:0-ACP) [12]. In Lauraceae family, the MCFA-specific FATBs have been identified and characterized in *Umbellularia californica*, *Cinnamomum Camphor*, *Cinnamomum longepaniculatum* and *Lindera communis* [13–16]. The residues or domains that are presumably responsible for the substrate specificities of these FATBs were also studied by site directed mutagenesis and domain swapping experiments [14, 17, 18]. Nevertheless, owing to the lack of information pertaining to FATB homologs from the LCFA-rich species in the family Lauraceae, a major knowledge gap that limiting our understanding of the molecular mechanisms for the drastic differences in the FA composition among different tree species in this family, has yet to be addressed.

In this study, we analyzed the seed oil content and FA composition of eleven species of the family Lauraceae. We obtained the evidence that seeds of *N. caudatum* contained high quantity of 18:2 and 18:0. Transcriptome analysis on its developing seeds was subsequently conducted to identify candidate genes involving in the LCFA formation and triacylglycerol biosynthesis in this species. Furthermore, the heterologous expression analysis reveals that NcFATB, which is structurally different from its counterpart from the MCFA-rich species, prefers long-chain saturated FAs. This is consistent with the richness of 18:0 in the seed of *N. caudatum*. Collectively, this study for the first time generated comprehensive molecular information regarding the seed oil biosynthesis in *N. caudatum*, thereby helping guide future efforts to manipulate oil production in certain tree species.

## Results

### The two fatty acids 18:2 and 18:0 occurred in high proportions in the seed of *N. caudatum*

The aim of our initial study was to identify tree species as a potential source for biofuel production. Hence, we analyzed the total lipid content and FA composition of collected seeds from 11 species of the family Lauraceae by gas chromatography. It was found that in the eight tree species *C. camphora*, *U. californica*, *A. forrestii*, *L. cubeb*, *L. communis, L. angustifolia*, *P. Americana* and *N. caudatum*, seed oil content reaches more than 25% on the dry weight basis versus less than 5% in *M. yunnanensis*, *P. cavaleriei* and *C. tonkinensis*. Furthermore, six of the above mentioned oil-rich species, i.e. *C. camphora*, *U. californica*, *A. forrestii*, *L. cubeb, L. angustifolia* and *L. communi*s, produce seed oils consisting predominantly of MCFA. In contrast, the seed oils of *P. Americana* and *N. caudatum* are mainly composed of LCFA, and the respective fatty acid composition is very similar to that found in three other tested species, i.e. *M. yunnanensis*, *P. cavaleriei* and *C. tonkinensis* (Table 1). Notably, *N. caudatum* appeared to have the highest proportion of 18:0 among all tested species (more than 20% of total FAs) (Table 1). A very interesting discovery is that although the FA composition of seed oil of *N. caudatum* is very similar to that of *C. tonkinensis*, a close relative species of *N. caudatum* [5], seed oil content of *N. caudatum* was much higher than that of *C. tonkinensis* (Table 1).

**Table 1:** Seed oil content and fatty acid composition of eleven species from the Lauraceae family

Species	Oil Content (% DW )	Capric acid 10:0 (%)	Lauric acid 12:0 (%)	Palmitic acid 16:0 (%)	Stearic acid 18:0 (%)	Oleic acid 18:1 (%)	Linoleic acid 18:2 (%)	Linolenic acid 18:3 (%)	Other (%)
*Cinnamomum camphor*	60.1±8.7	54.36±2.12	38.69±1.29	0.4±0.08	0.33±0.12	3.78±0.62	0.7±0.1	0.03±0.01	1.71±0.02
*Umbellularia californica*^a^[37]	40	27.5	60	2	0	5	3	0	2.5
*Actinodaphne forrestii*	35±4.01	8.18±1.12	71.7±9.12	1.87±0.34	0.44±0.09	4.66±0.32	11.23±0.98	0.23±0.04	1.45±0.23
*Litsea cubeba*	43.2±4.87	4.87±0.89	89.45±7.34	0.49±0.05	0.06±0.01	2.27±0.34	0.79±0.01	0.05±0.008	2.01±0.31
*Lindera communis*	31±2.7	14.44±2.32	57.71±8.48	3.63±0.56	0.61±0.04	11.99±1.21	4.13±0.43	0.56±0.2	6.93±0.89
*Lindera angustifolia*^a^[41]	38.8	55.5	29.6	1.1	0.2	9.3	1.7	0	2.2
*Persea americana*	47±4.1	0	0	22.53±1.43	6.53±0.42	28.99±1.64	36.3±2.31	1.32±0.32	4.32±0.43
*Machilns yunnanensis*	3.01±2.65	0.4±0.02	3.75±0.43	19.45±2.34	3.3±0.45	42.04±5.43	24.85±2.65	2.67±0.32	3.55±0.44
*Phoebe cabaleriei*	2.2±0.27	7.45±0.46	6.46±0.74	17.66±2.45	2.31±0.34	20.46±3.45	40.89±6.89	3.37±0.45	1.4±0.21
*Neocinnamomum caudatum*	45±3.7	0	0	9.28±1.01	20.77±2.21	15.81±1.51	37.89±4.32	11.76±1.23	4.48±0.43
*Caryodaphnopsis tonkinensis*	3±0.4	0	0	18.34±2.01	11.17±1.24	11.30±1.32	54.99±6.64	4.21±0.52	0

^a^Data were collected from the published literatures

**Fig. 1:**
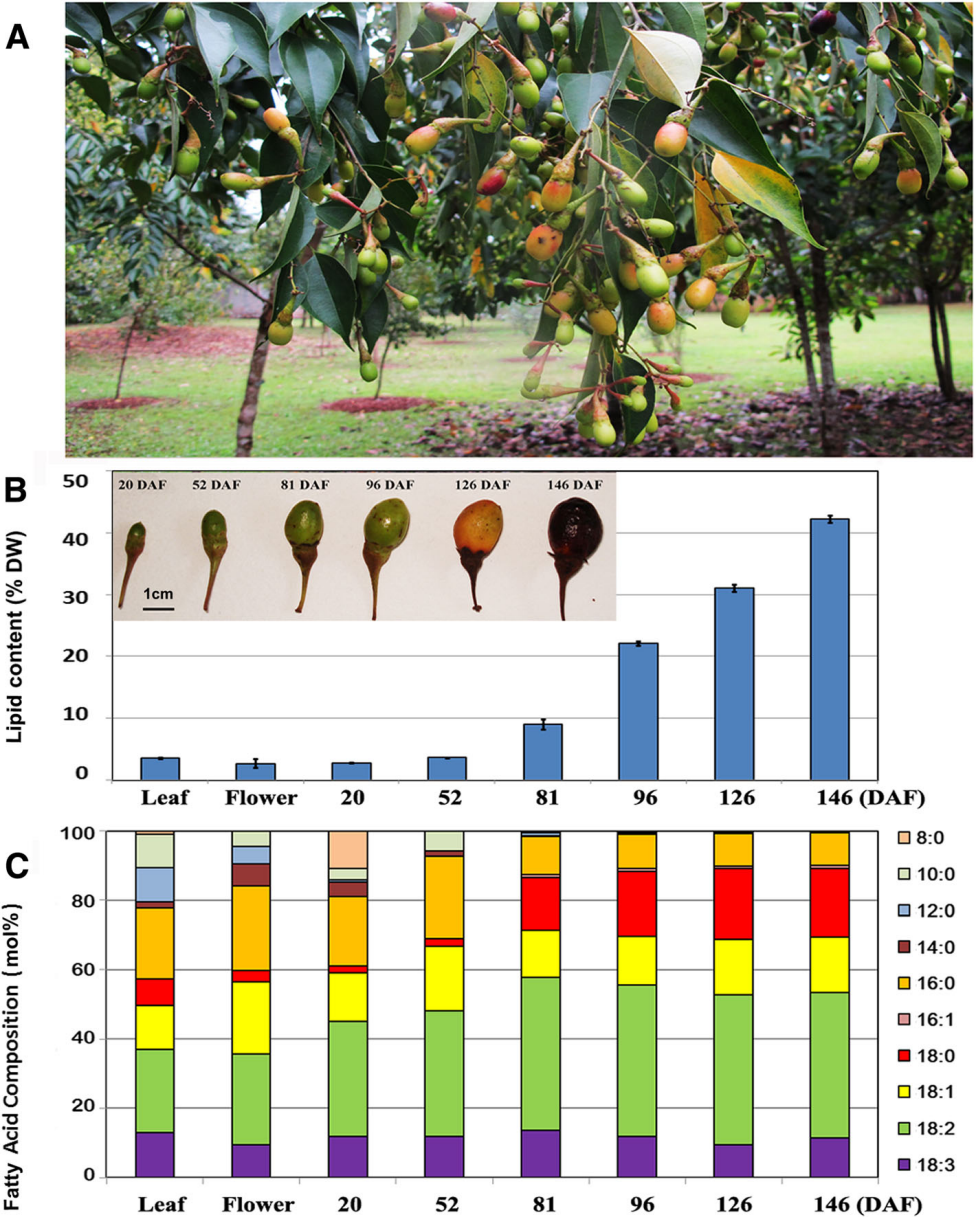
Total lipid content and FA composition in different tissues of *N. caudatum.* **a** *N. caudatum* fruits at the ripe season. **b** The lipid content of leaves, flowers and developing fruits (seed kernels) (20, 52, 81, 96, 126 and 146 days after flowering, DAF). (**c**) The fatty acid compositions of different tissues as shown in **b**. Data are means ± SD of three biological replications

To further understand lipid metabolism in *N. caudatum*, different tissues of three *N. caudatum* trees growing at Xishuangbanna Tropical Botanical Garden were collected at various developmental stages, and the total lipid content and fatty acid composition were analyzed (Fig. 1a). As shown in Fig. 1b, the fruits expanded rapidly after flowering, with the average diameter of fruits reaching 0.5 and 1.0 cm at 52 and 96 days after flowering (DAF), respectively. After 96 DAF, the size of most fruits stop expanding and the color of seed capsule started to turn red from green. In parallel, lipid analysis showed that the leaves and flower buds as well as developing seeds at the early stage (20 and 52 DAF) contained a limited amount of extracted lipids (only 2 to 4% on the dry weight basis) (Fig. 1b, Additional file 1). The predominant FAs in the seeds at 20 and 52 DAF were found to be 18:2 and 16:0, and their total quantity accounted for 40~59 % of the total FAs. As the embryos continued to develop, seed oil biosynthesis was accelerated, and the oil content elevated to 9, 22, 31 and 42 % on the dry weight basis at 81, 96, 126 and 146 DAF, respectively (Fig. 1b). Concomitantly, the FA composition also changed remarkably. While 18:2 constituting 40 % was still a major FA at 81 and 126 DAF, 16:0 declined significantly. Meanwhile, 18:0 increased up to 15 and 20 % of total FAs. In addition, the proportion of polyunsaturated fatty acids (PUFA, 18:2 and 18:3) was peaked at 81 DAF, followed by a slight decrease from 57 to 53 % of total FAs at 146 DAF (Fig. 1c).

### RNA-Seq, *de novo* assembly of unigenes and functional annotation

To discern the molecular mechanisms by which lipid metabolism is regulated in *N. caudatum*, its seeds at three development stages were used for RNA-Seq analysis. As a result, a total of 4,034 million paired-ends reads in nine libraries were generated, which account for 60.5 giga base pairs (GB) data (Table 2). Q20 and GC percentage of the reads in the libraries are also listed in Table 2. After eliminating the reads of adapters and low quality reads, 417 to 474 million clean reads were obtained from each sample. From the high quality clean reads, 529,269 contigs with an average length of 284 bp were *de novo* assembled. Meanwhile, 239,703 unigenes with an average length of 436 bp were obtained (Table 2). The size fraction of the unigenes showed that 52.5 % (125,761) of the unigenes had an average length less than 300 bp and only 1.4 % (3,417) of the unigenes were longer than 2000 bp (Additional file 2A).

**Table 2:** Summary of the sequencing data and transcriptome assembly of *N. caudatum*

Sample name	Reads Number	Bases(bp)	Q20%	Q30%	GC%	Clean Reads%	Clean data%
NCG3	42,453,220	6,367,983,000	96.200%	96.750%	52.348%	98.25%	97.55%
NCG3-1	44,398,646	6,659,796,700	98.410%	97.010%	55.522%	99.52%	97.86%
NCG3-2	47,613,018	7,141,952,700	98.630%	92.400%	55.249%	99.62%	97.82%
NCG4	41,465,990	6,219,898,500	96.450%	96.710%	56.525%	98.33%	97.53%
NCG4-1	43,498,292	6,524,743,800	98.440%	96.400%	57.546%	99.42%	97.77%
NCG4-2	44,286,814	6,643,022,100	98.370%	92.700%	57.552%	99.51%	97.81%
NCG5	45,271,522	6,790,728,300	96.230%	97.030%	55.130%	99.02%	95.26%
NCG5-1	47,142,326	7,071,348,900	98.670%	97.230%	58.820%	99.56%	97.37%
NCG5-2	47,270,260	7,090,539,000	98.810%	92.700%	57.479%	99.66%	97.82%
	Contig	Transcript	Unigene				
Sequence Number	529,269	292,915	239,703				
Total Length (bp)	150,731,679	145,105,800	104,677,564				
Max. Length (bp)	35,993	35,993	35,993				
Mean Length (bp)	284.7921926	495.3853507	436.6969291				
N50 (bp)	313	599	465				
N50 Sequence No.	113,058	59,215	54,084				
N90 (bp)	147	238	230				
N90 Sequence No.	402,463	226,680	191,049				
GC%	46.5%	46.23%	46.91%				

The gene annotation showed that 77,909 (32.5%), 43,261 (18.1%), 5,720 (2.4%), 73,195 (30.5%) and 77,131 (32.2%) unigenes were annotated to Nr, GO, KEGG, eggNOG and Swiss-Prot databases, respectively (Additional file 3). Notably, according to the BLASTX results in the Nr database, *N. caudatum* unigenes showed 25.3% (19,706), 7.7% (6,018), 7.3% (5,655) and 6.1% (4,758) sequence homologies to *Vitis vinifera*, *Populus trichocarpa*, *Cucumis sativus* and *Ricinus communis*, respectively (Additional file 2B). Besides, the E-value distribution of the top BLAST hits showed that 8.0% (19,267) of the unigenes had strong homology to previously deposited sequences (<1.0E^-60^), and 24.5% unigenes ranged from 1.0E^-5^ to 1.0E^-60^ (Additional file 2C). Likewise, the distribution of sequence identity showed that only 1.0 % (2,299) of the unigenes had high sequence similarity (95-100%) to the published sequences and 28.4% (68,107) had a sequence similarity less than 40% (Additional file 2D).

In GO classification, a total of 43,261 predicted proteins were annotated to biological processes (38.2%), molecular functions (39.4%) and cellular components (22.5%) (Additional file 4). In the biological processes category, cellular process (26.9%) and metabolic process (26.5%) were the predominant groups, followed by single-organism process (18.1%), biological regulation (5.6%), localization (5.5%), response to stimulus (5.0%) and cellular component organization or biogenesis (4.2%). In the cellular components category, cell (22.3%) and cell part (22.2%) were the most dominant groups, followed by organelle (15.7%), membrane (13.8%) and membrane part (8.8%). Meanwhile, the molecular function category included catalytic activity (44.2%), binding (42.8%), transporter activity (5.1%) and structural molecule activity (3.7%).

For eggNOG classification [19], 22.3 and 16.4 % of 90,178 unigenes were categorized into general function predicted only and function unknown, followed by posttranslational modification, protein turnover, chaperones (6.6%), signal transduction mechanisms (6.2%), replication, recombination and repair (4.9%), ribosomal structure and biogenesis (4.9%), and transcription (4.6%) (Additional file 5). Notably, only 2.49% of unigenes (2,248) were annotated to lipid transport and metabolism.

To explore the metabolic functions and interactions of the detected unigenes, we analyzed the detected unigenes using a pathway based analysis KEGG (Kyoto Encyclopedia of Genes and Genome) [20]. A total number of 5,374 unigenes were assigned to 35 groups of five major categories: metabolism, genetic information processing, environmental information processing, cellular processes and organismal systems. The major pathways were related to translation (556 unigenes, 10.3%), carbohydrate metabolism (410 unigenes, 7.6%) and signal transduction (409 unigenes, 7.6%) (Additional file 6). Notably, 237 unigenes (4.4%) were annotated to lipid related metabolisms, including FA biosynthesis, glycerolipid metabolisms, linoleic acid metabolism and α-linolenic acid metabolism (Additional file 7).

### Identification of unigenes associated with FA formation and triacylglycerol biosynthesis through DEG analysis

**Fig. 2:**
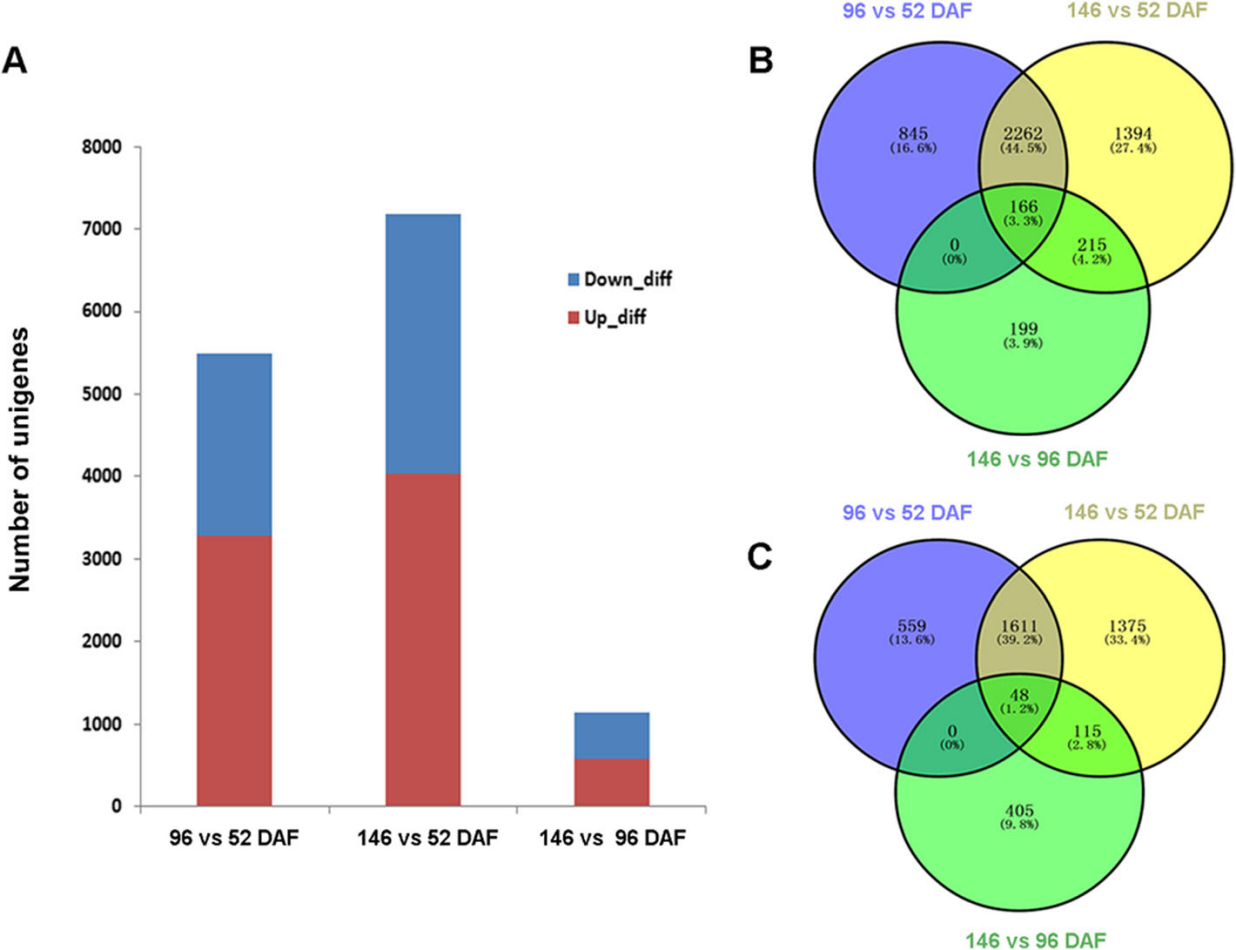
Number and distribution of DEGs in developing *N. caudatum* seeds. **a** The number of up or down-regulated unigenes during *N. caudatum* seed development. **b** The distribution of up-regulated genes at different seed developmental stages. **c** The distribution of down-regulated genes at different seed developmental stages

Since the oil content and FA composition of *N. caudatum* seed vary greatly with its developmental stages, DEG analyses were performed between each two of three fruit developmental stages to understand the mechanisms underlying the changes of oil content and FA composition. Three developmental stages correspond to 52, 96 and 146 DAF. In brief, a total of 5,493, 7,183 and 1,150 unigenes were found differentially expressed in 96 vs. 52 (Contrast Group I), 146 vs. 52 (Contrast Group II) and 146 vs. 96 DAF (Contrast Group III), respectively (Fig. 2). More specifically, 3,274, 4,038 and 581 unigenes were up-regulated, and 2,219, 3,150, and 569 unigenes were down-regulated within each contrast group, respectively (Fig. 2a). As shown by the Venn diagram, 166 unigenes were also co-expressed for the up-regulated genes in each contrast group. In addition, 845, 1,394 and 199 upregulated unigenes were specific to Contrast Group I, II and III, respectively (Fig. 2b). As for the down-regulated unigenes, 48 unigenes were co-expressed in all three contrast groups, and 559, 1,375 and 409 down-regulated unigenes were specific to Contrast Group I, II and III, respectively (Fig. 2c).

To understand the function of these DEGs, GO classification and KEGG pathway enrichment were performed. According to the GO analysis, 5,493, 7,188 and 1,150 DEGs in Contrast Group I, II and III were assigned to 44 subsets of three major categories (Additional file 8). In the cellular component category, the most dominant subsets included cell part (34.5%, 15.2% and 17.4% of total DEGs in each contrast group, respectively), membrane (21.1%, 8.7% and 11.1%) and intracellular (18.3%, 7.1% and 7.1%). In the biological process category, cellular metabolic process (20.2%, 5.1 % and 6.1%), primary metabolic process (20.2%, 5.1 % and 6.6%) and macromolecule metabolic process (13.4 %, 3.4 % and 3.0 %) were the primary subsets. Moreover, in the molecular function category, transferase activity (11.2%, 3.6% and 4.1%), hydrolase activity (8.7%, 2.7% and 4.0%), nucleotide binding (8.1%, 2.2% and 1.0%) and ion binding (7.5%, 2.3% and 3.6%) were the most prevailing subsets (Additional file 8). Based on the KEGG analysis, 1,228, 1,435 and 312 DEGs in the three contrast groups were assigned to 239, 249 and 112 KEGG pathways, respectively. Notably, 54, 71 and 17 DEGs were annotated to the lipid metabolism in all three contrast groups, respectively (see Additional file 9). More specifically, the Fragments Per Kilobase of Exon per Million Fragments Mapped (FPKM) values of two stearoyl-ACP desaturase (SAD) unigene (c87644_g1 and c79724_g1) changed remarkably along with fruit maturation: the FPKM value of c87644_g1 was higher at the early stage of fruit development (52 DAF) while c79724_g1 was highly expressed at the late stage (Additional file 10). Likewise, unigenes that were annotated to glycerol-3-phosphate acyltransferase (GPAT), 3-oxoacyl-[acyl-carrier-protein] synthase II, 3-oxoacyl- [acyl-carrier protein] reductase, diacylglycerol acyltransferase (DGAT) and lyso-phosphatidaylcholine acyltransferase (LPCAT) were all differentially expressed at different stages of fruit maturation (Additional file 10).

**Fig. 3:**
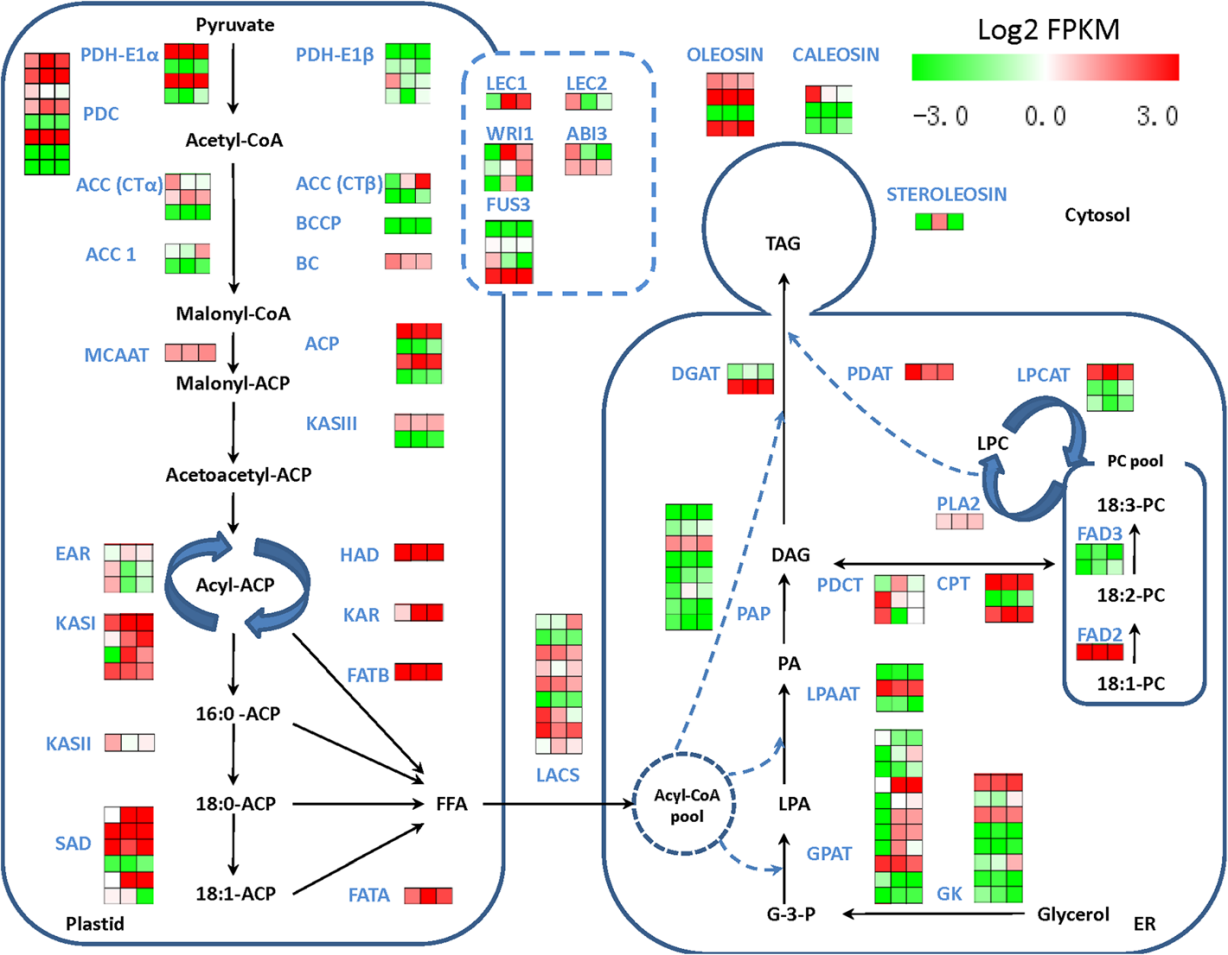
Predicted pathways of FA formation and triacylglycerol biosynthesis in *N. caudatum* developing seeds. The FPKM values of the 137 unigenes were log2 transformed and demonstrated with colored boxes in the FA formation and TAG biosynthesis pathways. Three boxes in the horizontal direction represented for Log2 transformed FPKM values of NCG3-1, NCG4-1 and NCG5-1 seed samples. Boxes in the vertical direction represent for numbers of homologous unigenes identified by the Local Blast. Abbreviations: PDH, pyruvate dehydrogenase; a-CT, alpha subunit of heteromeric ACCase; β-CT, beta subunit of heteromeric ACCase; BC, biotin carboxylase of heteromeric ACCase; BCCP, biotin carboxyl carrier protein of heteromeric ACCase; MCAAT, malonly-CoA ACP transferase; KASI/II/III, ketoacyl-ACP synthase I/II/III; KAR, 3-ketoacyl-ACP reductase; HAD, 3-hydroxyacyl-ACP dyhydratase; EAR, 2-enoyl-ACP reductase; SAD, stearoyl-ACP desaturase; FATA/B, Acyl-ACP thioesterase A/B; LACS, long-chain acyl-CoA synthase; FAD2, Δ12 desaturase; FAD3, Δ15 (ω-3) desaturase; GPAT, glycerol 3-phosphate acyltransferase; LPAAT, lysophosphatidic acid acyltransferase 2; PAP, phosphatidic acid phosphatase; PDCT, phosphatidylcholine: diacylglycerol cholinephospho transferase; CPT, diacylglycerol cholinephospho transferase; PLA2, phospholipase A2; LPCAT, lysophosphatidylcholine acyltransferase; DGAT, diacylglycerol acyltransferase; PDAT, phospholipid: diacylglycerol acyltransferase; ACP, acyl carrier protein; *LEC1/2, LEAFY COTYLEDON 1/2*; *WRI1, WRINKLED 1*; *ABI3, ABSCISIC ACID INSENSITIVE 3*; *FUS3, FUSCA 3*; G-3-P, glycerol-3-phosphate; LPA, lysophosphatidic acid; PA, phosphatidic acid; LPC, lysophosphatidylcholine; PC, phosphatidylcholine; DAG, diacylglycerol; TAG, triacylglycerol

**Fig. 4:**
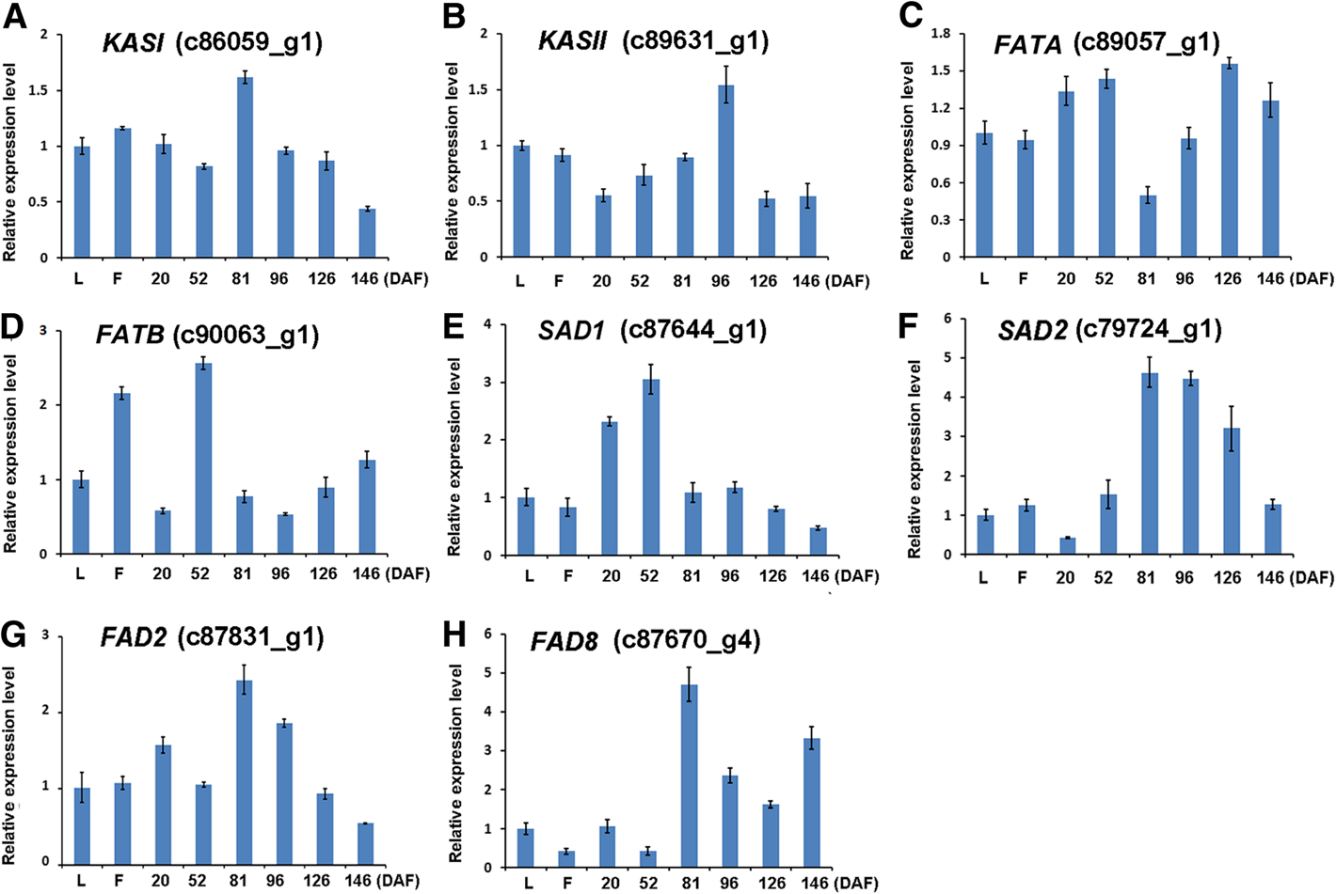
Validation of gene expression levels by qRT-PCR. The expression levels of *KASI*  (**a**), *KASII* (**b**), *FATA* (**c**), *FATB* (**d**), *SAD1* (**e**), *SAD2* (**f**), *FAD2* (**g**) and *FAD8* (**h**) in the leaves (L), flowers (F) and developing fruits (20, 52, 81, 96, 126 and 146 DAF) were validated with qRT-PCR. *ACT11* was selected as the reference gene. Values are means ± SD (normalized that the expression levels in the leaves equal to 1) of three biological replications

Based on the local-blast search against the database with 81 key *Arabidopsis* genes that are involved in FA formation and triacylglycerol synthesis (Additional file 10), 137 unigenes were identified with high sequence similarities (E-value < 1.0E 10^-5^) to the *Arabidopsis* homologs. Among these 137 identified unigenes, 15 of them were predicted to code for pyruvate dehydrogenase complex (PDHC), 9 for ACCase, 5 for SAD and 4 for FAD8. In contrast, only a single homolog encoding individual FAD2/3/6/7 enzyme or FATA/B was identified in the *N. caudatum* genome (Additional file 10). According to the log2 transformed FPKM of these unigenes (Fig. 3), *FATB, SAD, FAD2* and *FAD8* genes displayed much higher expression levels than did *FATA*, *KASII* and *FAD3*. Moreover, qRT-PCR analysis revealed that *KASI, KASII, FATA, FATB, SAD1, SAD2, FAD2* and *FAD8* genes were expressed in all of the tissues tested, including leaves, flowers and developing fruits. Overall, the expression patterns of these genes coincided with the results from RNA-Seq analysis (Fig. 4 a-h). The transcripts of *KASII*, *FAD2, FAD8* and *SAD2* were accumulated at higher levels during the stages of rapid oil synthesis (81-96 DAF) and then decreased drastically as the fruit ripens. In contrast, the expression levels of *FATB* and *SAD1* were relatively high at the early embryo development stage (52 DAF) and decreased rapidly during fruit maturation (Fig. 4). Interestingly, the expression of *FATA* in the fruits dropped to its lowest level at 81 DAF and then started to increase along with fruit maturation (146 DAF), which was agreed with 18:1 accumulation in the seeds.

### Functional characterization of NcFATB in *E. coli* BL21 (DE3) strain

**Fig. 5:**
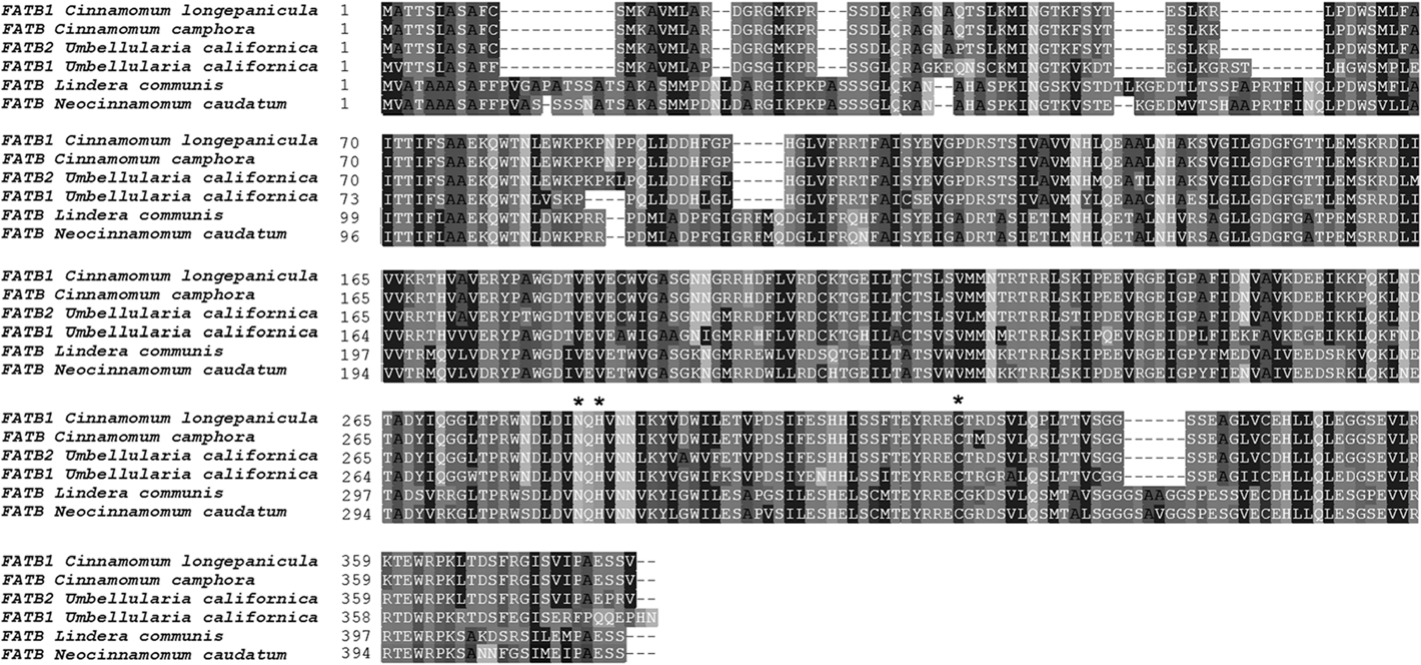
Alignment of FATB protein sequences among species of the Lauraceae family. The deduced polypeptides sequence of NcFATB was aligned with FATB protein sequences of *Lindera communis* (AHF72806), *Cinnamomum camphora* (AAC49151), *Umbellularia californica* (AAC49001 & AAA34215) and *Cinnamomum longepaniculatum*. The conserved catalytic residues in the C-terminus were marked with asterisk

**Fig. 6:**
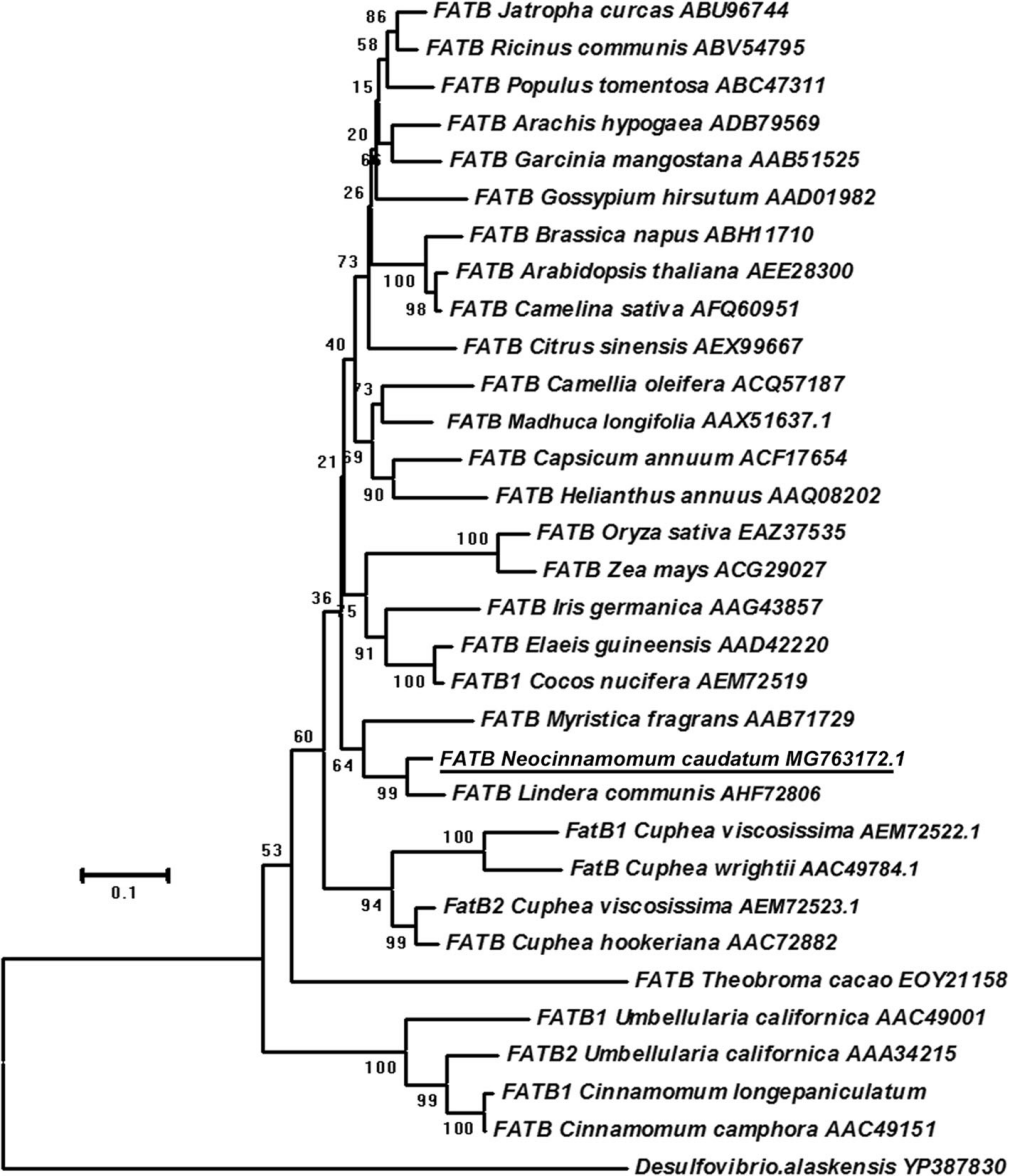
Phylogenetic analysis of NcFATB protein sequences in various species. A neighbor-joining tree based on 31 FATB protein sequences form 29 plant species was constructed in Mega 6.0. YP387830 protein in *Desulfovlbrlo alaskensis* was selected as the out group

In this study, only one unigene (ID: c90067_g1) annotated as *FATB* was identified in *N. caudatum* developing seeds. Its transcripts were shown to accumulate at detectable levels in all of the tested fruit samples. The full-length CDS of this unigene, designated *NcFATB*, was subsequently cloned and sequenced. The deduced NcFATB peptide has 416 amino acid residues with the molecular weight of 45.87 kDa and the theoretical pI of 6.29. Multiple sequence alignment revealed that NcFATB shares 90, 81 and 80% similarity with its counterpart from *L. communis* (AHF72806), *U. californica* (AAC49001) and *C. camphora* (AAC49151), respectively (Fig. 5). Asn^312^, His^314^ and Cys^348^ located in the C terminus of NcFATB could be the presumed catalytic residues, as suggested in other FATB proteins [14, 21]. The phylogenetic analysis showed that NcFatB is evolutionally more close to the LCFA acyl-ACP catalyzing FatBs rather than the MCFA acyl-ACP catalyzing FatBs such as CcFatB, UcFatB and ClFatB (Fig. 6). The phylogenetic analysis also suggested that NcFatB may prefer LCFA acyl-ACP as substrates.

**Fig. 7:**
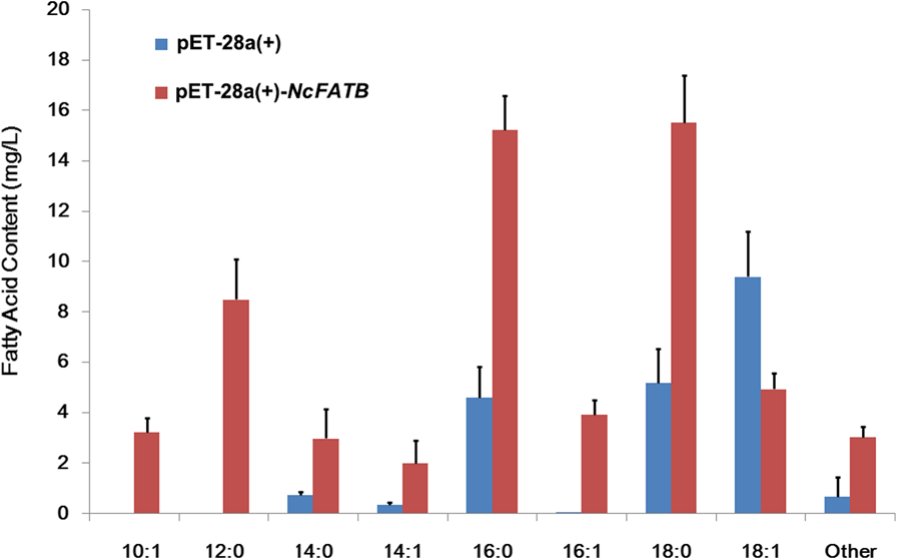
Free FAs detected in *NcFATB*-expressing *E. coli* BL21 (DE3) culture medium. *E. coli* BL21 (DE3) harboring pET-28a(+)-*NcFATB* expression vector or blank pET-28a(+), were induced with 0.1 mM IPTG at 30 °C for a time course of 24 hours. Free FAs in bacterial medium were extracted, methyl esterified and analyzed with GC-FID. Values presented are means ± SD of three biological replicates

To experimentally determine its function, *NcFATB* under the control of a T7 promoter was heterologously expressed in *E.* coli BL21(DE3). Five hours after IPTG induction at various concentrations (0, 0.1, 0.5 and 1 mM) at 30°C, a clear band near 50 kD was detected by SDS-PAGE (Additional file 11), which is corresponding to the NcFATB protein in the supernatant of bacterial lysate. At 24 hours after IPTG induction, the total FAs in the cell culture expressing NcFATB were increased up to 59.3 mg/L versus 21 mg/L for that of the pET-28a(+) empty vector. There was also an obvious difference in the FA composition between these different cell lines (Fig. 7). In the supernatant of *NcFATB*-expressing cell culture, the amounts of 16:0 and 18:0 were changed most dramatically, increasing from approximately 4.6 and 5.2 mg/L to 15.2 and 15.5 mg/L, respectively. On the contrary, the content of 18:1 in the supernatant of this line (4.9 mg/L) decreased compared with that of the control cells (9.4mg/L). In addition, decenoic acid (10:1, 3.2±0.5 mg/L), lauric acid (12:0, 8.5±1.6 mg/L), myristic acid (14:0, 3.0±1.0 mg/L), myristoleic acid (14:1, 2.0±1.0 mg/L) and hexadecenoic acid (16:1, 3.9±0.6 mg/L) were detected in the medium of *NcFATB*-expressing cell culture (Fig. 7). Our data suggest that NcFATB possesses a high capacity to release long-chain saturated FAs (16:0 and 18:0) from the respective acyl-ACP substrates.

## Discussion

The exploitation of new plant resources for their oil-producing characteristics as well as for less demand for farmland has received much attention over the past 30 years [19, 20, 22]. China is rich in wild oil-producing plant resources. More than 400 Lauraceae species have been discovered in China [6]. Many of them were found dominant in MCFA in their seeds [7, 23]. These MCFA lipids could serve as indispensable raw materials for numerous industrial products, such as surfactants, lubricants and detergents [6, 24]. In this study, *C. camphora*, *U. californica*, *A. forrestii*, *L. cubeb* and *L. communis* were discovered to contain relatively high seed oil content (>30% DW) and the dominant FAs in these seeds were exclusively MCFA (Table 1). In contrast, LCFA was dominant in the seeds of *N. caudatum, L. angustifolia*, *P. americana*, *M. yunnanensis*, *P. cavaleriei* and *C. tonkinensis*. Strikingly, the FAs of *N. caudatum* seeds were mainly composed of 18:2 and 18:0, which is remarkably different from that of other well-documented Lauraceae species such as *C. camphora* and *U. californica* (Table 1). To our knowledge, not many species in the plant kingdom have been reported to exhibit such unique seed oil profile [25–27]. In light of this, we speculated that a distinct mechanism for lipid metabolism may occur in *N. caudatum* and the study of this specific molecular mechanism may enable us to identify the target plants suitable for future genetic engineering for seed oil production.

RNA-Seq is an effective means to study the molecular regulation of a particular trait in a new plant species that lacks any reference genomes [19, 28, 29]. Recent advances in this technology have made it relatively easy to identify critical genes and pathways associated with lipid metabolism in some Lauraceae species such as *C. camphor* [30], *Litsea cubeba* [31], *Persea americana* [32, 33] and *Lindera glauca* [34]. Given that little genomic information about *N. caudatum* is currently available, we initially conducted high-throughput transcriptome sequencing of developing seeds of this species. The data from the RNA-Seq experiment combined with subsequent validation by qRT-PCR would help generate a comprehensive picture about the possible causes of the characteristic fatty acid profile in the seed oil of *N. caudatum*.

In higher plants, the 18:0 level can be regulated by three types of enzymes [9]. The first type of enzyme is the plastid-localized KASII, which catalyzes the elongation of 16:0-ACP to 18:0-ACP [35]. As shown in a previous study, overexpression of *KASII* gene in sunflower seeds could elevate the 18:0 levels significantly [34]. Unexpectedly, the FPKM values of *NcKASII* were relatively low in the seeds of *N. caudatum* (Additional file 10). In agreement, the qRT-PCR results showed that the expression level of *KASII* decreased remarkably at the fruit maturation stage (126 DAF), which is an important stage for 18:0 accumulation (Fig. 4b). Nevertheless, it remains unclear at this stage whether NcKASII is a key determinant of the high 18:0 accumulation in *N. caudatum* seeds. The second type of enzyme controlling the 18:0 levels is FAT, which releases FAs from acyl-ACP. It was discovered that the FPKM value of *NcFATB* was much higher than that of *NcFATA* in all the developing seeds tested (Additional file 10). Accordingly, RT-PCR showed that the expression levels of *NcFATB* gradually increased after 81 DAF (Fig. 4d), which were correlated with the 18:0 levels in the seeds (Fig. 1c), suggesting that the *NcFATB* gene may play an important role in controlling 18:0 accumulation in *N. caudatum* seeds. The third type of enzyme is SAD, which catalyzes the conversion of 18:0-ACP to 18:1-ACP in the plastids. The formed 18:1-ACP is subjected to hydrolysis exerted primarily by FATA [9]. Since the total amount of 18:1, 18:2 and 18:3 are higher than that of 18:0 in *N. caudatum* seed lipid, it can be inferred that high NcSAD activities are present in the developing seeds. This view is supported by the high FPKM values of *NcSAD1* and *NcSAD2* (Fig. 3, Additional file 10). Nevertheless, the results from DEG analysis and qRT-PCR showed that the expression levels of *NcSAD1* and *NcSAD2* varied greatly among different stages during seed development (Fig. 4e & f). The two *NcSAD* genes displayed opposite expression patterns, suggesting that they may play distinct roles in FA accumulation (Fig. 4). Last, it should be pointed out that although it is unknown to what extent the plastidial acyltransferases play a role in the control of the 18:0 levels in the seed oil, such a role cannot be excluded since they may compete for the same substrate 18:0-ACP with NcFATB and NcSAD enzymes.

It is generally accepted that *FAD2* is the key determinant of the 18:2 level in oilseeds. In agreement with this, the FPKM values of *NcFAD2* in developing seeds were very high (>400, Additional file 10), which were correlated with the high level of this FA in the seeds. Nevertheless, the expression levels of *NcFAD2* varied with the different stages of seed development (Fig. 4g). Interestingly, the FPKM value of *NcFAD8* (107670_g4) coding for a plastidal desaturase was shown to be much higher than that of *NcFAD3* (Additional file 10)*.* In addition, the high level of *NcFAD8* expression, as shown by qRT-PCR, seems to coincide with the relatively high level of 18:3 accumulation in the developing seeds (Fig. 1 and Fig. 4h). This intriguing result may raise a question as to whether the conversion of 18:2 to 18:3 by this enzyme significantly contributes to the final level of 18:3 in the seed oil. Such a question merits further investigation.

In this study, we also found the deduced amino acid sequence of NcFATB shared high sequence homology with UcFATB1/2, CcFATB and LcFATB (Fig. 5). It belongs to the FAT family (PF01643) and contains a helix/multi S stranded sheet motif (hotdog folds) with three conserved catalytic residues in the C terminus (N^312^, H^314^ & C^348^) [21, 36]. Interestingly, like LcFATB, NcFATB has 35-37 extra residues compared to CcFATB, UcFATB and ClFATB (Fig. 5). Those extra residues appear to be conserved and are distributed in the N and C termini of NcFATB protein (Fig. 5). Considering the fact that the seeds of *U. californica, C. camphor* and *C. longipaniculatum* contain predominantly MCFA, while *L. communis* sarcocarp and *N. caudatum* seeds are rich in LCFA [16, 37], there is a possibility that these extra 35-37 residues in the FATB sequence may influence the substrate specificity. Our phylogenetic analysis also showed the protein sequence of NcFATB and LcFATB were grouped together and shared close relationships with the LCFA-dominant species. In contrast, three MCFA-specific FATBs were grouped together and spread away from other LCFA-specific FATBs (Fig. 6). In the future studies, site-directed mutagenesis or domain swapping of NcFATB may help define the roles of individual residues in controlling the substrate preference of this protein.

Heterologous expression of target gene in *E. coli* cells has proven to be an effective way to determine gene function [38]. The substrate specificities of FATB could be inferred from the contents of free FAs in the culture medium, as reported in a previous study where expression of *UcFATB* in *E. coli* FA degradation mutant strain K27 resulted in huge elevation of MCFA (more than 80 % of total FAs) in the medium [39]. In this study, we found that heterologous expression of NcFATB in *E. coli* BL21 (DE3) cells resulted in a 2.86-fold increase in total free FAs. LCFAs (16:0, 18:0, 16:1 and 18:1) constituted 66.7% of the total FAs in the culture medium, whereas the two MCFAs, 10:1 and 12:0, only reached 20% of the total FAs (Fig. 7). Since the amount of free LCFAs is 3.37-fold higher than that of free MCFAs in the culture medium, it is reasonable to speculate that NcFATB prefers LCFA-ACP over MCFA-ACP as substrates, which is consistent with the fact that the seed of *N. caudatum* only has a very limited amount of MCFAs. As to the low-level accumulation of free MCFA in the medium of bacterial culture, one probable explanation is that the high-level expression of NcFATB protein in the BL21 (DE3) cells may trigger weak hydrolysis of MCFA-ACP even if this enzyme has low affinity for MCFA-ACP (Additional file 11). This assumption is supported by a previous study with FATB of *L. communis* whose sarcocarps contain predominantly LCFAs [39]. In this reported study, heterologous expression of LcFATB resulted in similar amount of MCFA accumulation in the culture medium of *E. coli* BL21 (DE3) *fad88* mutant strain [39]. Together, our results suggest that NcFATB prefers LCFA-ACP as the substrate although we cannot rule out the possibility that the heterologously expressed NcFATB may utilize MCFA-ACP as substrate to some extent, especially under some favorable conditions. To more accurately define the substrate preference of NcFATB, it is necessary to dissect the *in planta* function of this gene in the future.

## Conclusions

In this study, the lipid content and FA composition of eleven species from Lauraceae family were first quantified, and the transcriptome in the developing seeds of *N. caudatum* was analyzed. NcFATB was shown to possess a unique structure and a high capacity to use long-chain saturated fatty acyl-ACPs as substrates, providing an explanation for the 18:0-rich oil profile in *N. caudatum* seeds. Our study depicted a comprehensive view of triacylglycerol biosynthesis in *N. caudatum* seeds, which may be useful in exploiting this plant species as an industrial resource.

## Methods

### Plant materials and sampling

From Aug 2015 to Feb 2016, 100 g matured leaves, flower buds and developing fruits (labeled at 20, 52, 81, 96, 126 and 146 day after flowering, DAF) were collected separately from 3 individual *N. caudatum* trees at Xishuangbanna Tropical Botanical Garden, Yunnan Province, China*.* Meanwhile, the matured seeds of *Cinnamomum camphora* (L.) J.Presl, *Actinodaphne forrestii* (C.K.Allen) Kosterm., *Litsea cubeba* (Lour.) Pers., *Lindera communis* Hemsl., *Persea americana* Mill., *Machilns yunnanensis* Lecomte, *Phoebe cabaleriei* (H. Lé v.) Y. Yang et Bing Liu and *Caryodaphnopsis tonkinensis* (Lecomte) Airy Shaw were also harvested in the same location (samples of each species were collected from 3 individual trees, see Additional file 12). The samples were immediately frozen in liquid nitrogen and stored at -80°C till lipid analysis and RNA extraction.

### Lipid analysis

To analyze the lipid content and FA composition, seed kernels from nine species of Lauraceae family were collected and grinded into fine powders in liquid nitrogen. Lipid extraction was performed as previously described [40]. Glyceryl triheptadecanoate (Cat# T2151, Sigma-Aldrich, USA) was added as internal standard (50 μg each sample). The extracted lipids were esterified into FA methyl esters (FAMEs) and analyzed with GC-FID (Agilent 7890B Gas Chromatography equipped with DB23 column, 60m*0.25mm* 0.25 μm, Agilent,USA). The temperature program initiated with 160°C for 1.5 minutes and increased to 240°C at a rate of 20°C/minutes, then kept at 240°C for 10 minutes. The total lipid content and FA compositions were calculated by comparing the peak area of target FAs and the internal standard (methyl heptadecanoate). Data presented are mean ± SD of three biological replicates. The seed oil content and FA composition of *U. californica* and *L. angustifolia* were taken from previous published literatures for comparison purposes [37, 41].

### RNA extraction and cDNA library construction

Three important stages of *N. caudatum* fruits, early cotyledon development stage (52 DAF), fast oil accumulating stage (96 DAF) and fully maturation stage (146 DAF) from three individuals, were selected for RNA-seq analysis [2]. Total RNA of the fruits was extracted by using the RNeasy Plant Mini Kit (Qiagen, USA). DNase I (RQ1, Promega, USA) was added to remove any genomic DNA contamination. Total RNA was quantified using Nanodrop ND-2000 spectrophotometer (Nanodrop Technologies, USA). All the samples showed a 260/280 nm ratio of 1.8 to 2.1. The poly-A tailed mRNA was purified from the total RNA using Dynabeads™ mRNA Purification Kit (Cat # 61006,Thermo Fisher Scientific, USA). The first-strand cDNA fragments were synthesized with random primers and transformed into double-strand cDNA. Fragments of desirable lengths (200-300 bp) were purified, end-repaired and ligated with the sequencing adapters through A and T complementary base pairing. The sequencing library was constructed using polymerase chain reaction (PCR). The synthesized cDNA libraries were normalized to a 10 nM and gradually diluted and quantified to 4-5 pM.

### Deep sequencing, unigenes assembly and gene annotation

Nine cDNA libraries were deep-sequenced on the Illumina NextSeq™ 500 platform at Shanghai Personal Biotechnology Co., Ltd. A total of 424 - 476 million paired-ends raw reads in each library were sequenced. After filtering out the low quality reads (mean mass fraction<Q20) and raw reads with adaptors, 417- 474 million clean reads in each library were *de novo* assembled using Trinity software (Version: r20140717, k-mer 25 bp) [42]. With default parameters and 25-mer k-mer size, 239,703 unigenes were assembled. The unigene sequences were then aligned using BLASTx against the NR (NCBI non-redundant protein sequences), eggNOG (evolutionary genealogy of genes: Non-supervised Orthologous Groups, Version4.0) and Swiss-Prot databases with an E-value cutoff of 10^-5^ [43]. GO (Gene Ontology) functional classification and KEGG (Kyoto Encyclopedia of Genes and Genome) annotation were performed with BLAST2GO [42] and KASS [44] (KEGG Automatic Annotation Server, http://www.genome.jp/tools/kaas/).

### Differential expression gene(DEG)analysis

DEG screening was conducted by DESeq (Version 1.18.0) [45]. Genes were estimated to be significantly differentially expressed if expression values showed a log2 (fold change) >1 and P value < 0.05 between any two tested developmental stages. Co-expression analysis of DEG and Venn diagram were drawn by VENNY2.1 (http://bioinfogp.cnb.csic.es/tools/venny/index.html). GO functional categorization of DEGs was performed with BGI WEGO program [46] (http://wego.genomics.org.cn /cgi-bin/wego/index.pl). The ten most-represented GO terms of each category were demonstrated in figures. KEGG annotation of DEGs was performed on KASS as described above.

### Identification and expression analysis of unigenes

To identify the putative genes that associated with long-chain saturated FA formation and triacylglycerol biosynthesis, a local-blast search was performed between *N. caudatum* unigenes and 81 *Arabidopsis* genes which were crucial for FA formation and triacylglycerol assembling [47]. Unigenes that had high sequence similarity (E value < 10^-5^) to the *Arabidopsis* homologs were identified (Additional file 10). Besides, their Fragments Per Kilobase of transcript per Million (FPKM) values were log2 transformed and demonstrated with Multi Experiment Viewer (MeV) software. Moreover, the expression levels of *FATA/B, FAD2/8, KASI/II* and *SAD1*/*2* in the samples were further validated with qRT-PCR (CFX96 Real Time PCR System, Bio-red, USA; SYBR Premix Ex Taq™ Cat # RR420, TaKaRa, Japan). The primers were designed based on the conserved sequences of these unigenes (See Additional file 13) and *ACT11* was selected as the reference gene. The relative expression level of each target gene was calculated by delta-delta Cq method [48] and normalized with its expression level in the leaves (equal to 1 ). Three biological replicates were conducted and data were presented as mean ± SD.

### Cloning and heterologous expression of *NcFATB*

The full-length CDS of *NcFATB* was amplified from the cDNA of *N. caudatum* seeds. The forward primer (5’- ATGGTTGCCACCGCTGCTGCTTC -3’) and the reverse primer sequences (5’- CTATGAGCTCTCAGCTGGAATCTCCATG -3’) were suggested by transcriptomic sequencing results. The amplified sequence was cloned into pMD19-T vector (Cat# 3271, Takara, Japan) and verified by Sanger sequencing. The deduced protein sequence of *NcFATB* was ClustalW multiple aligned with five published FATBs from Lauraceae species in Bioedit software (version 7.9.9.0). The neighbor-joining (NJ) tree was constructed based on the poisson model in MEGA6.06 (Version: 6140226). To characterize its function, the full-length CDS of *NcFATB* was codon optimized and chemically synthesized (Genscript Co. Ltd., China, see Additional file 14). The sequence was then sub-cloned into pET-28a (+) (Cat# 69864-3, Novagen, Germany) expression vectors (with *NdeI* and *XhoI* digestion sites) and transformed into *E. coli* BL21 (DE3) competent cells. The recombinant cells were inoculated into 200 ml LB medium (containing 50 μg/ml kanamycin, amended with 15 g/L glucose as supplementary carbohydrate), and cultured at 37 °C, 180 rpm. When OD_600nm_ reached 0.4, 100 mM isopropyl-β-D- thiogalactopyranoside (IPTG) was added to the final concentration of 0.1 mM. The bacterial cells were then cultivated for another 24 hours at 30 °C [49]. Bacterial cultures were centrifuged at 4000 rpm at 4 °C for 10 minutes. The supernatants were then collected for free FA analysis as previously described [21]. A bacterial cell transformed with empty pET-28a (+) vector was cultured in parallel as control. The expression of NcFATB was monitored by SDS-polyacrylamide gel electrophoresis (PAGE) as previously described [16].

## Additional files

Additional file 1: Lipid analysis of *N. caudatum* tissue samples*.* The total lipid content and FA compositions of leaves, flowers and developing fruits (20, 52, 81, 96, 126 and 146 days after flowering, DAF) were quantified with GC-FID. Data are means ± SD of three biological replications. (DOCX 19 kb)

Additional file 2: Length distribution of unigenes in *N. caudatum* and homology search against NR database. (A) Length distribution of *N. caudatum* unigene. (B) Species distribution of top BLASTX hits of *N. caudatum* with other plant species in Nr database. (C) E-value distribution of best BLASTX hits in Nr database. (D) Distribution of sequence identity of unigenes with BLAST hits in Nr database. (TIF 654 kb)

Additional file 3: Summery of *N. caudatum* unigene functional annotations in different databases. The unigenes were blastX with NR, GO, KEGG, eggNOG and Swissprot database. (TIF 2123 kb)

Additional file 4: Functional classification of Gene ontology (GO) annotation of *N. caudatum* unigenes. Unigenes were assigned into three categories: biological process, cellular components and molecular functions. (TIF 771 kb)

Additional file 5: Evolutionary genealogy of genes: Non-supervised Orthologous Groups (eggNOG) classification of *N. caudatum* unigenes. A: RNA processing and modification. B: Chromatin structure and dynamics. C: Energy production and conversion. D: Cell cycle control, cell division, chromosome partitioning. E: Amino acid transport and metabolism. F: Nucleotide transport and metabolism. G: Carbohydrate transport and metabolism. H: Coenzyme transport and metabolism. I: Lipid transport and metabolism. J: Translation, ribosomal structure and biogenesis. K: Transcription. L: Replication, recombination and repair. M: Cell wall/membrane/envelope biogenesis. N: Cell motility. O: Posttranslational modification, protein turnover, chaperones. P: Inorganic ion transport and metabolism. Q: Secondary metabolites biosynthesis, transport and catabolism. R: General function prediction only. S: Function unknown. T: Signal transduction mechanisms. U: Intracellular trafficking, secretion, and vesicular transport. V: Defense mechanisms. W: Extracellular structures. X: Undetermined. Y: Nuclear structure. Z: Cytoskeleton. (TIF 1237 kb)

Additional file 6: KEGG annotations of *N. caudatum* unigenes. KEGG annotation was performed with a web-based tool KASS (KEGG Automatic Annotation Server, http://www.genome.jp/tools/kaas/). (TIF 1682 kb)

Additional file 7: Functional annotations of unigenes in *N. caudatum* developing seeds. (XLSX 15909 kb)

Additional file 8: GO classification of DEGs in the developing seeds of *N. caudatum*. GO analysis of DEGs was performed by BGI WEGO online platform (Web Gene Ontology Annotation Plot, http:// wego.genomics.org.cn/cgi-bin/wego/index.pl). Ten most representative subsets of each functional category were presented. (TIF 882 kb)

Additional file 9: KEGG annotation of DEGs in the developing seeds of *N. caudatum*. KEGG annotations of DEGs among three contrast groups were performed with KASS). (XLSX 133 kb)

Additional file 10: Identification of unigene that involved in lipid metabolisms by Local Blast. Based on the local-blast search against the database with 81 key *Arabidopsis* genes that are involved in FA formation and triacylglycerol synthesis, we identified 137 unigenes that have high sequence similarities (E-value < 1.0E 10^-5^) to the *Arabidopsis* homologs. (XLSX 27 kb)

Additional file 11: SDS-PAGE of NcFATB protein in *E. coli* BL21 (DE3) bacterial cells. Five hours after IPTG induction (0, 0.1, 0.5 & 1 mM), the pET-28a(+)-*NcFATB* transformed bacterial cells were washed, re-suspended and sonificated in Tris-HCl (pH=7.5). After centrifugation, the supernatants of bacterial lysate were loaded on the SDS-PAGE gel. The lysate of bacterial cells transformed with pET-28a (+) empty vector was loaded as control (CK). (TIF 477 kb)

Additional file 12: Sampled species of Lauraceae and their voucher specimens in this study. (DOCX 30 kb)

Additional file 13: Gene-specific primer sequences for qRT-PCR validation. (DOCX 16 kb)

Additional file 14: The codon-optimized sequence of *NcFATB*. The full-length CDS of *NcFATB* was codon optimized and chemically synthesized in Genscript Co. Ltd. (China) for further validation of gene function in *E. coli* BL21 (DE3) cells. (DOCX 14 kb)

## Abbreviations

*ABI3*: *ABSCISIC ACID INSENSITIVE 3*
ACP: Acyl carrier protein
a-CT: Alpha subunit of heteromeric ACCase
BC: Biotin carboxylase of heteromeric ACCase
BCCP: Biotin carboxyl carrier protein of heteromeric ACCase
CPT: Diacylglycerol cholinephospho transferase
DAG: Diacylglycerol
DEG: Differentially Expressed Gene
DGAT: Diacylglycerol acyltransferase
EAR: 2-enoyl-ACP reductase
EggNOG: Evolutionary genealogy of genes: Non-supervised Orthologous Groups
FAD2: Δ12 desaturase
FAD3: Δ15 (ω-3) desaturase
FAs: Fatty acids
FATA/B: Acyl-ACP thioesterase A/B
FPKM: Fragments Per Kilobase of transcript per Million fragments mapped
FUS3: FUSCA 3
G-3-P: Glycerol-3-phosphate
GO: Gene Ontology
GPAT: Glycerol 3-phosphate acyltransferase
HAD: 3-hydroxyacyl-ACP dyhydratase
KAR: 3-ketoacyl-ACP reductase
KASI/II/III: Ketoacyl-ACP synthase I/II/III
KEGG: Kyoto Encyclopedia of Genes and Genome
LACS: Long-chain acyl-CoA synthase
LCFA: Long-chain FA
LPA: Lysophosphatidic acid
LPAAT: Lysophosphatidic acid acyltransferase 2
LPC: Lysophosphatidylcholine
LPCAT: Lysophosphatidylcholine acyltransferase
MCAAT: Malonly-CoA ACP transferase
MCFA: Medium-chain FA
PA: Phosphatidic acid
PAP: Phosphatidic acid phosphatase
PC: Phosphatidylcholine
PDAT: Phospholipid: diacylglycerol acyltransferase
PDCT: Phosphatidylcholine: diacylglycerol cholinephospho transferase
PDH: Pyruvate dehydrogenase
PLA2: Phospholipase A2
PUFA: Polyunsaturated FA
SAD: Stearoyl-ACP desaturase
TAG: Triacylglycerol
*WRI1*: *WRINKLED 1*
β-CT: Beta subunit of heteromeric ACCase
*ABI3*: *ABSCISIC ACID INSENSITIVE 3*
ACP: Acyl carrier protein
a-CT: Alpha subunit of heteromeric ACCase
BC: Biotin carboxylase of heteromeric ACCase
BCCP: Biotin carboxyl carrier protein of heteromeric ACCase
β-CT: Beta Subunit of heteromeric ACCase
CPT: Diacylglycerol cholinephospho transferase
DAG: Diacylglycerol
DEG: Differentially Expressed Gene
DGAT: Diacylglycerol acyltransferase
EAR: 2-enoyl-ACP reductase
EggNOG: Evolutionary genealogy of genes: Non-supervised Orthologous Groups
FAs: Fatty acids
FAD2: Δ12 desaturase
FAD3: Δ15 (ω-3) desaturase
FATA/B: Acyl-ACP thioesterase A/B
FPKM: Fragments Per Kilobase of transcript per Million fragments mapped
FUS3: FUSCA 3
GO: Gene Ontology
GPAT: Glycerol 3-phosphate acyltransferase
G-3-P: Glycerol-3-phosphate
HAD: 3-hydroxyacyl-ACP dyhydratase
KAR: 3-ketoacyl-ACP reductase
KASI/II/III: Ketoacyl-ACP synthase I/II/III
KEGG: Kyoto Encyclopedia of Genes and Genome
LACS: Long-chain acyl-CoA synthase
LCFA: Long-chain FA
*LEC1/2*: *LEAFY COTYLEDON 1/2*
LPA: Lysophosphatidic acid
LPAAT: Lysophosphatidic acid acyltransferase 2
LPC: Lysophosphatidylcholine
LPCAT: Lysophosphatidylcholine acyltransferase
MCAAT: Malonly-CoA ACP transferase
MCFA: Medium-chain FA
PA: Phosphatidic acid
PAP: Phosphatidic acid phosphatase
PC: Phosphatidylcholine
PDAT: Phospholipid: diacylglycerol acyltransferase
PDCT: Phosphatidylcholine: diacylglycerol cholinephospho transferase
PDH: Pyruvate dehydrogenase
PLA2: Phospholipase A2
PUFA: Polyunsaturated FA
SAD: Stearoyl-ACP desaturase
TAG: Triacylglycerol
*WRI1*: *WRINKLED 1*
8:0: Decanoic acid
10:0: Capric acid
12:0: Lauric acid
16:0: Palmitic acid
18:0: Stearic acid
18:1: Oleic acid
18:2: Linoleic acid
*LEC1/2*: *LEAFY COTYLEDON 1/2*
8:0: Decanoic acid
10:0: Capric acid
12:0: Lauric acid
16:0: Palmitic acid
18:0: Stearic acid
18:1: Oleic acid
18:2: Linoleic acid
18:3: linolenic acid
