# Supplementary material for: Transcriptome analysis reveals a composite molecular map linked to unique seed oil profile of Neocinnamomum caudatum (Nees) Merr
Source: BMC Plant Biol. 2018 Nov 26;18:303. doi: 10.1186/s12870-018-1525-9 (PMC6258453; doi:10.1186/s12870-018-1525-9)
Supplement: Supplementary file 1 — Lipid analysis of N. caudatum tissue samples. The total lipid content and FA compositions of leaves, flowers and developing fruits (20, 52, 81, 96, 126 and 146 days after flowering, DAF) were quantified with GC-FID. Data are means ± SD of three biological replications. (DOCX 19 kb) [file 12870_2018_1525_MOESM1_ESM.docx]

**Table S3 Lipid analysis of *N. caudatum* tissue samples**

| **Tissues** | **Lipid Content (%)** | **Capric acid**  **10:0 (%)** | **Lauric acid**  **C12:0 (%)** | **Palmitic acid**  **C16:0 (%)** | **Stearic acid**  **C18:0 (%)** | **Oleic acid**  **C18:1 (%)** | **Linoleic acid**  **C18:2 (%)** | **Linolenic acid**  **C18:3 (%)** | **Other**  **(%)** |
| --- | --- | --- | --- | --- | --- | --- | --- | --- | --- |
| Leaf | 3.58±0.09 | 9.60±0.43 | 10.01±0.89 | 20.32±2.21 | 7.68±0.56 | 12.77± 1.12 | 23.83±2.43 | 13.12±1.21 | 2.68±0.23 |
| Flower | 2.74±0.7 | 4.52±0.44 | 5.06±0.43 | 24.37±2.21 | 3.28±0.21 | 20.76±1.87 | 26.12±2.62 | 9.62±0.76 | 6.27 ±0.53 |
| Seeds 20 DAF | 2.83±0.1 | 3.39±0.34 | 0.62±0.08 | 20.01±2.88 | 2.03±0.19 | 13.9±1.22 | 33.28± 3.27 | 11.87±1.01 | 14.88 ±1.57 |
| Seeds 52 DAF | 3.67 ±0.07 | 5.72±0.52 | 0 | 23.73±2.12 | 2.30± 0.11 | 18.72±2.10 | 36.03±2.98 | 12.02±1.01 | 1.48±0.21 |
| Seeds 81 DAF | 9.09 ±0.8 | 0.19±0.02 | 0.98 ±0.12 | 11.04±0.89 | 15.12±1.23 | 13.56±1.12 | 44.20±3.89 | 13.67±1.42 | 1.23 ±0.22 |
| Seeds 96 DAF | 22.13 ±0.3 | 0.16±0.02 | 0.38±0.03 | 10.01±0.89 | 18.58±1.78 | 14.04±1.23 | 43.63±5.32 | 11.98± 1.27 | 1.21 ±0.11 |
| Seeds 126 DAF | 31.09±0.6 | 0 | 0.32±0.02 | 9.49±0.78 | 20.30±1.89 | 16.17± 1.45 | 43.20±3.34 | 9.48±1.01 | 1.05 ±0.09 |
| Seeds 146 DAF | 42.27 ±0.6 | 0 | 0.26±0.02 | 9.50±0.69 | 19.73±1.21 | 16.02±1.54 | 41.98±5.32 | 11.51±1.21 | 1.01±0.08 |
