# Supplementary figures and images for: Transcriptome analysis reveals a composite molecular map linked to unique seed oil profile of Neocinnamomum caudatum (Nees) Merr
Source: BMC Plant Biol. 2018 Nov 26;18:303. doi: 10.1186/s12870-018-1525-9 (PMC6258453; doi:10.1186/s12870-018-1525-9)

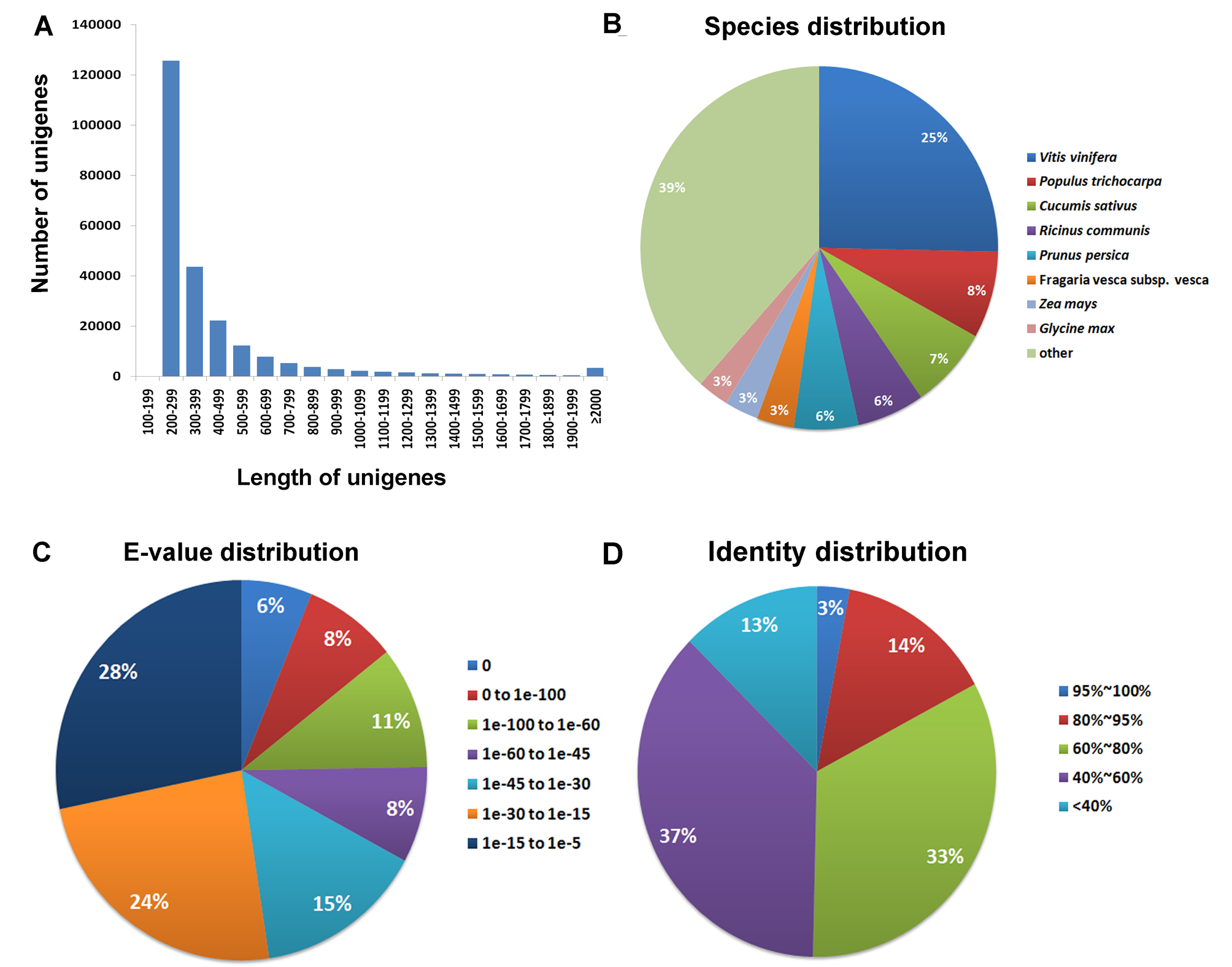

Supplement: Supplementary file 2 — Length distribution of unigenes in N. caudatum and homology search against NR database. (A) Length distribution of N. caudatum unigene. (B) Species distribution of top BLASTX hits of N. caudatum with other plant species in Nr database. (C) E-value distribution of best BLASTX hits in Nr database. (D) Distribution of sequence identity of unigenes with BLAST hits in Nr database. (TIF 654 kb) [file 12870_2018_1525_MOESM2_ESM.tif]

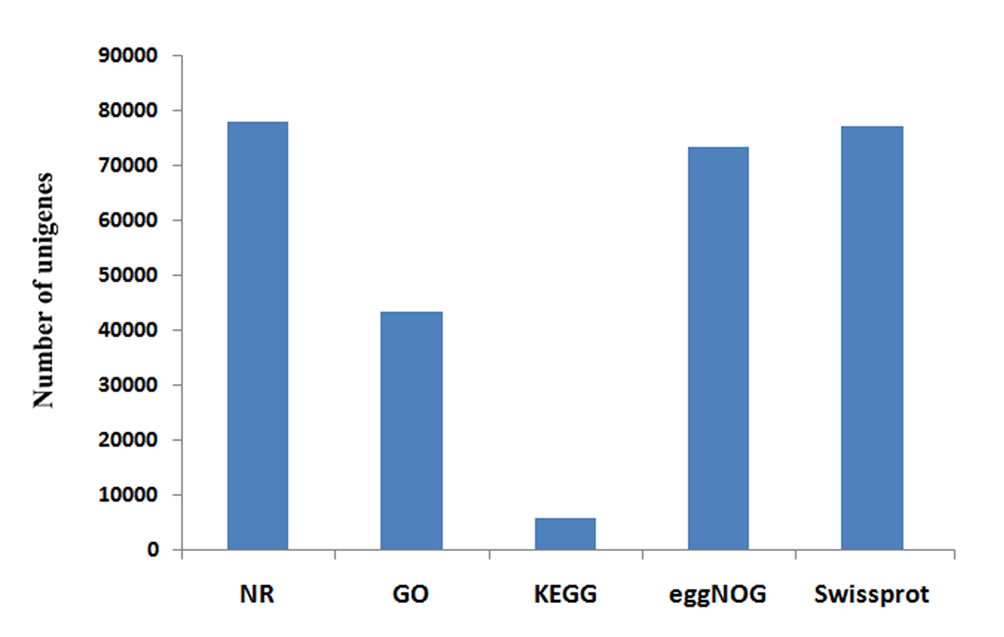

Supplement: Supplementary file 3 — Summery of N. caudatum unigene functional annotations in different databases. The unigenes were blastX with NR, GO, KEGG, eggNOG and Swissprot database. (TIF 2123 kb) [file 12870_2018_1525_MOESM3_ESM.tif]

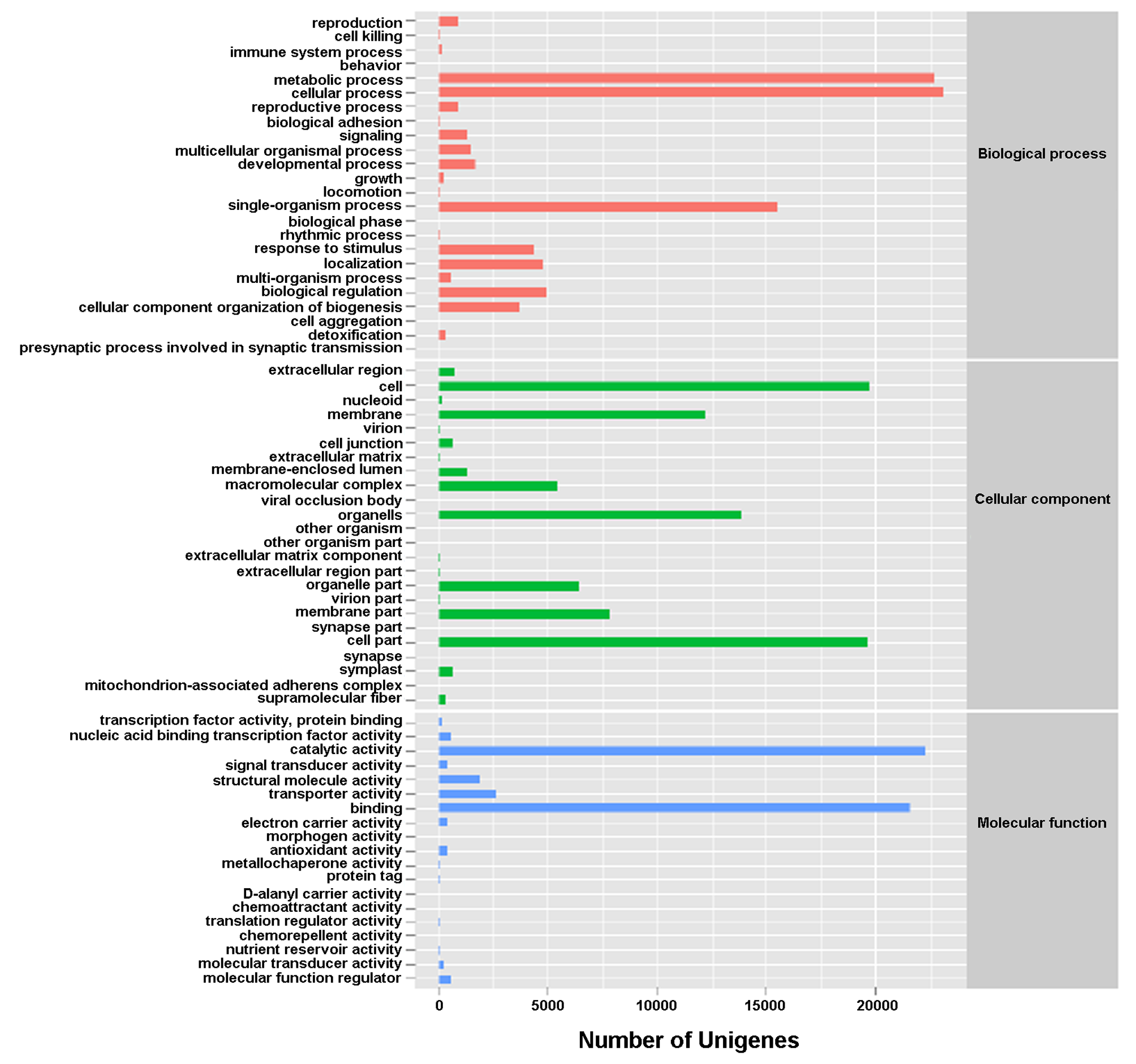

Supplement: Supplementary file 4 — Functional classification of Gene ontology (GO) annotation of N. caudatum unigenes. Unigenes were assigned into three categories: biological process, cellular components and molecular functions. (TIF 771 kb) [file 12870_2018_1525_MOESM4_ESM.tif]

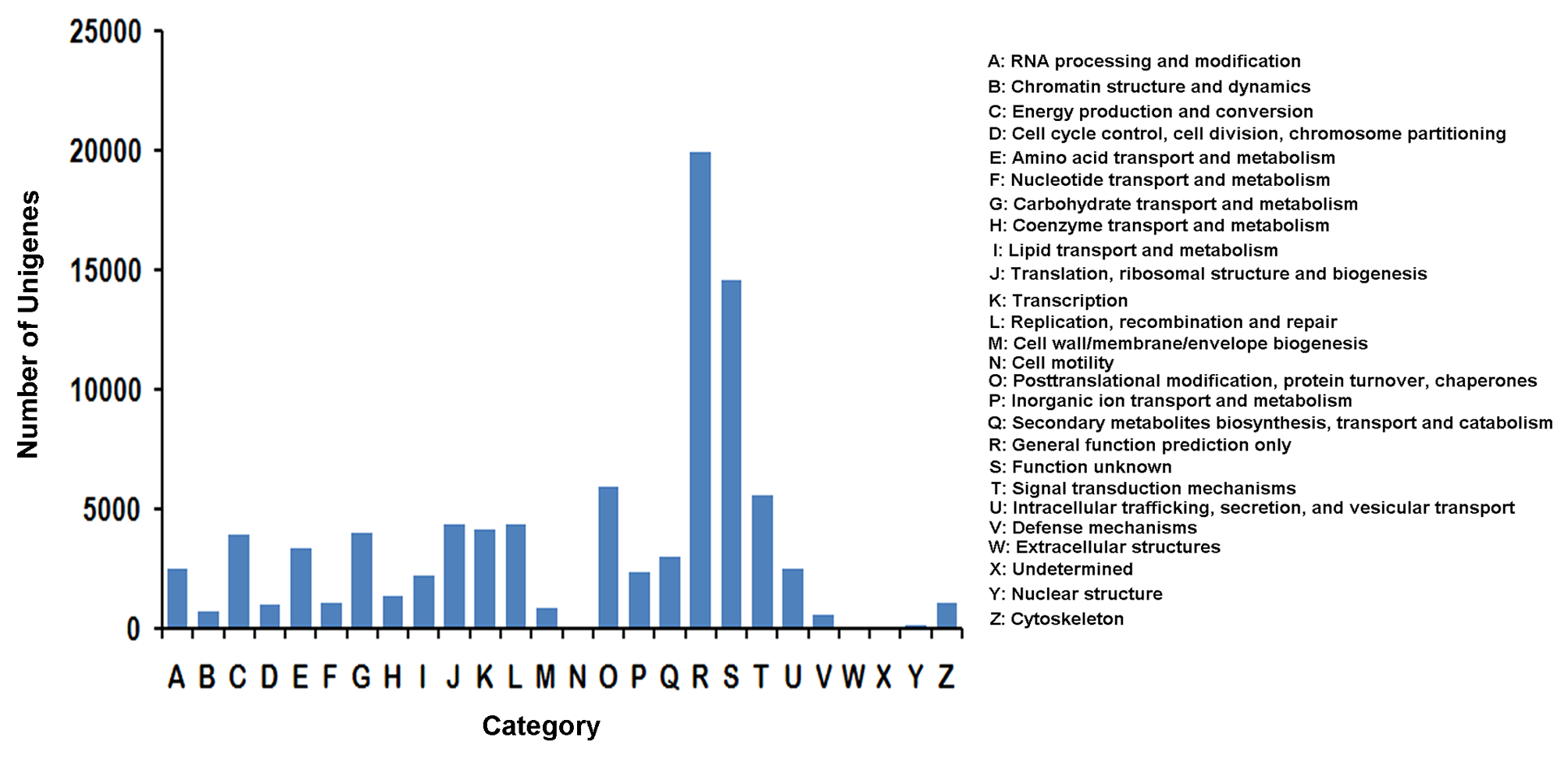

Supplement: Supplementary file 5 — Evolutionary genealogy of genes: Non-supervised Orthologous Groups (eggNOG) classification of N. caudatum unigenes. A: RNA processing and modification. B: Chromatin structure and dynamics. C: Energy production and conversion. D: Cell cycle control, cell division, chromosome partitioning. E: Amino acid transport and metabolism. F: Nucleotide transport and metabolism. G: Carbohydrate transport and metabolism. H: Coenzyme transport and metabolism. I: Lipid transport and metabolism. J: Translation, ribosomal structure and biogenesis. K: Transcription. L: Replication, recombination and repair. M: Cell wall/membrane/envelope biogenesis. N: Cell motility. O: Posttranslational modification, protein turnover, chaperones. P: Inorganic ion transport and metabolism. Q: Secondary metabolites biosynthesis, transport and catabolism. R: General function prediction only. S: Function unknown. T: Signal transduction mechanisms. U: Intracellular trafficking, secretion, and vesicular transport. V: Defense mechanisms. W: Extracellular structures. X: Undetermined. Y: Nuclear structure. Z: Cytoskeleton. (TIF 1237 kb) [file 12870_2018_1525_MOESM5_ESM.tif]

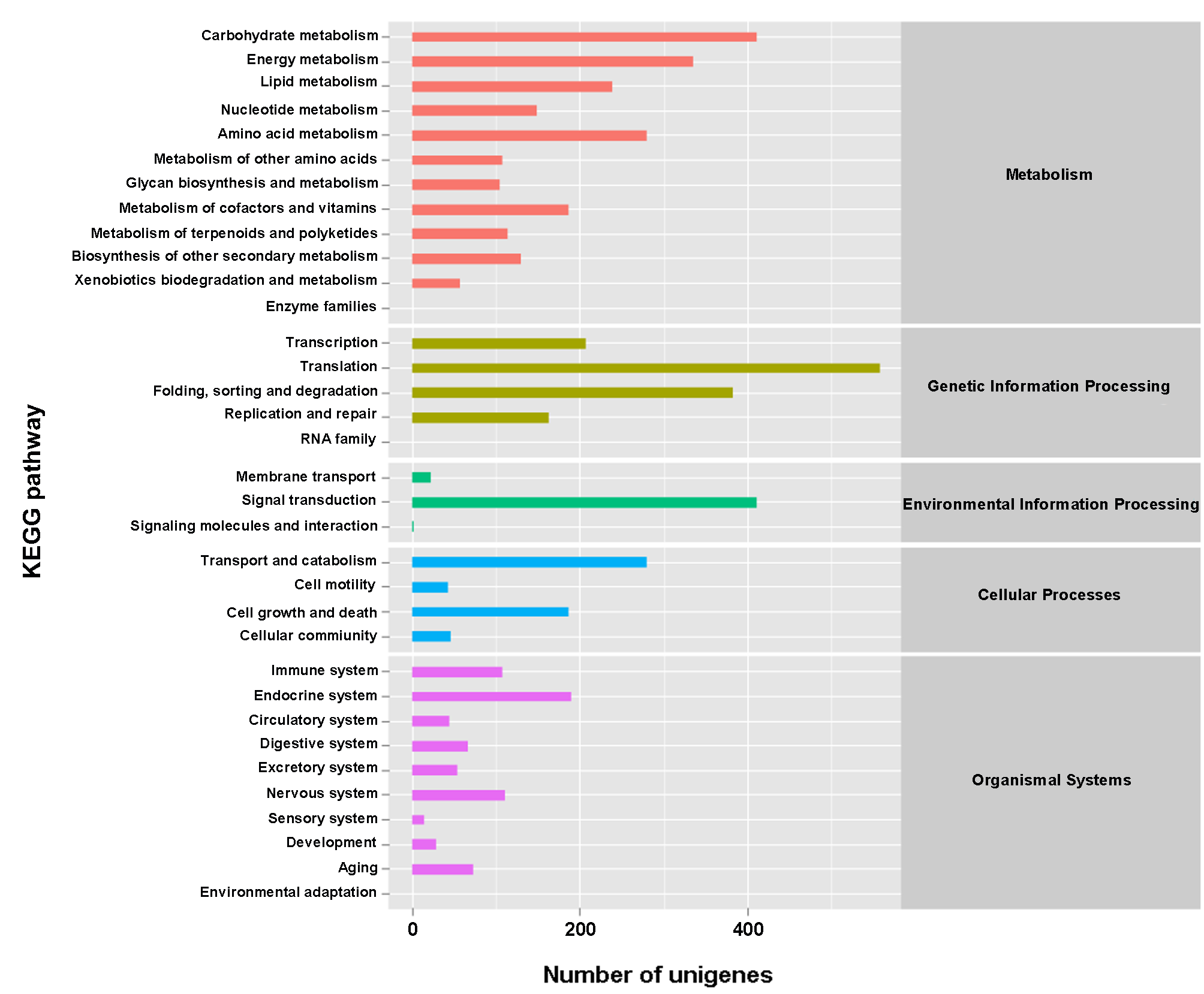

Supplement: Supplementary file 6 — KEGG annotations of N. caudatum unigenes. KEGG annotation was performed with a web-based tool KASS (KEGG Automatic Annotation Server, http://www.genome.jp/tools/kaas/). (TIF 1682 kb) [file 12870_2018_1525_MOESM6_ESM.tif]

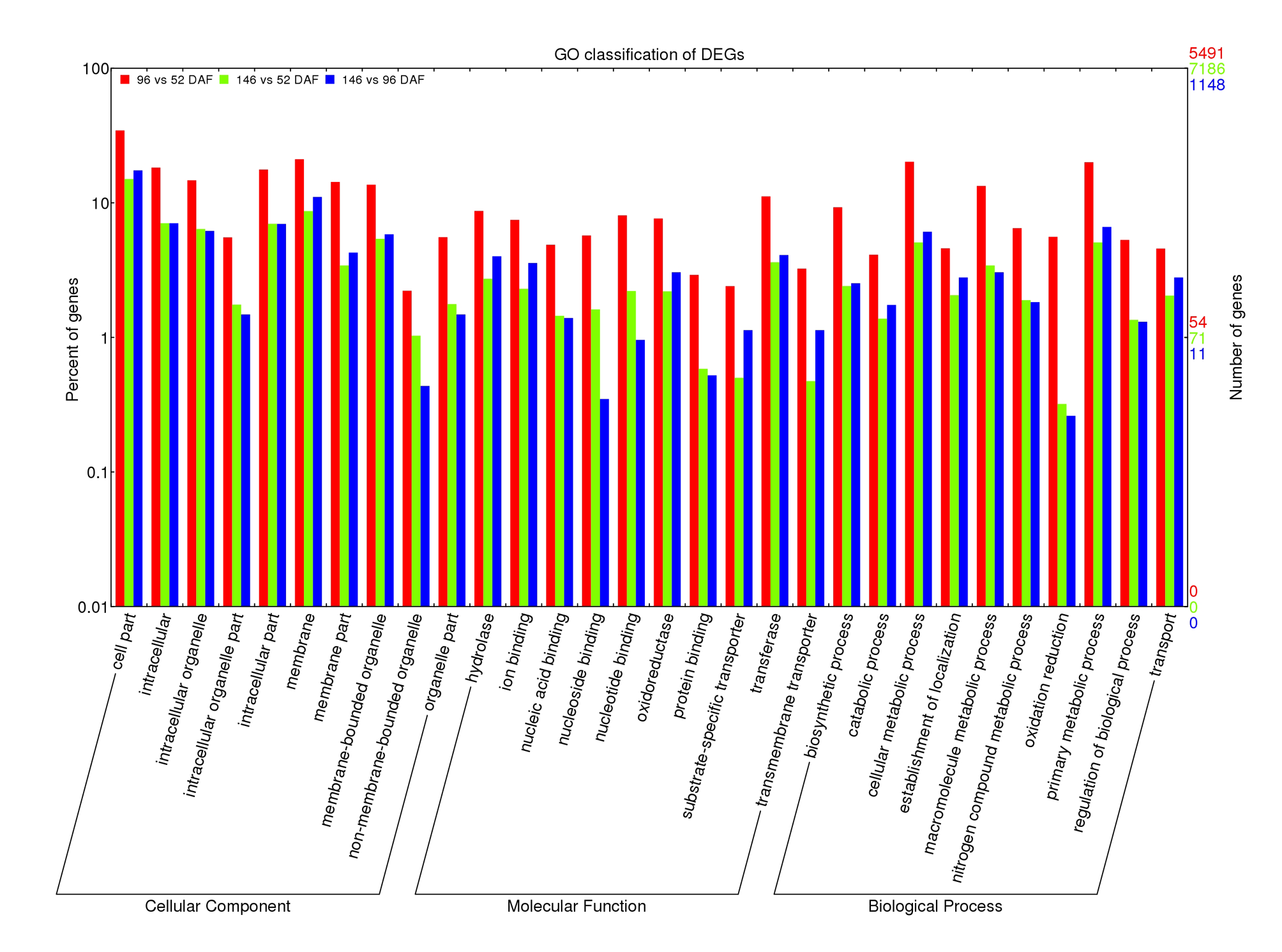

Supplement: Supplementary file 8 — GO classification of DEGs in the developing seeds of N. caudatum. GO analysis of DEGs was performed by BGI WEGO online platform (Web Gene Ontology Annotation Plot, http:// wego.genomics.org.cn/cgi-bin/wego/index.pl). Ten most representative subsets of each functional category were presented. (TIF 882 kb) [file 12870_2018_1525_MOESM8_ESM.tif]

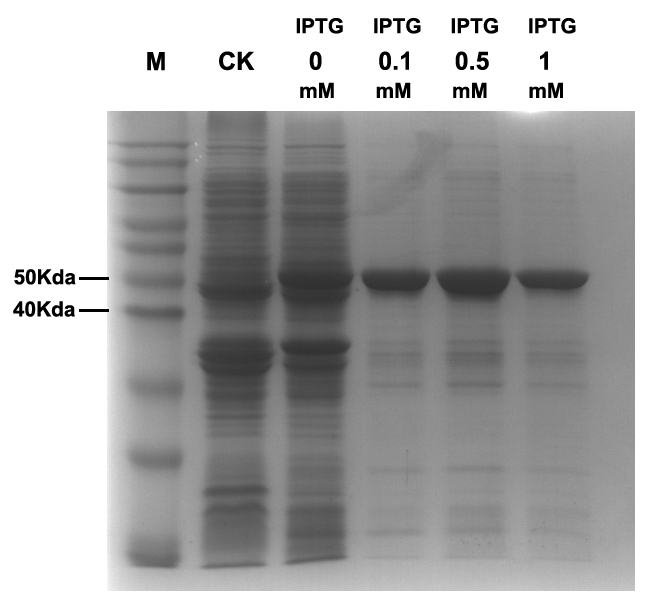

Supplement: Supplementary file 11 — SDS-PAGE of NcFATB protein in E. coli BL21 (DE3) bacterial cells. Five hours after IPTG induction (0, 0.1, 0.5 & 1 mM), the pET-28a(+)-NcFATB transformed bacterial cells were washed, re-suspended and sonificated in Tris-HCl (pH=7.5). After centrifugation, the supernatants of bacterial lysate were loaded on the SDS-PAGE gel. The lysate of bacterial cells transformed with pET-28a (+) empty vector was loaded as control (CK). (TIF 477 kb) [file 12870_2018_1525_MOESM11_ESM.tif]
