# Supplementary material for: Transcriptome analysis reveals a composite molecular map linked to unique seed oil profile of Neocinnamomum caudatum (Nees) Merr
Source: BMC Plant Biol. 2018 Nov 26;18:303. doi: 10.1186/s12870-018-1525-9 (PMC6258453; doi:10.1186/s12870-018-1525-9)
Supplement: Supplementary file 12 — Sampled species of Lauraceae and their voucher specimens in this study. (DOCX 30 kb) [file 12870_2018_1525_MOESM12_ESM.docx]

| **Table S1. Sampled species of Lauraceae and their voucher specimens in this study.** | | | | | |  |  |
| --- | --- | --- | --- | --- | --- | --- | --- |
| **No** | **Taxon** | **Herbarium** | **Voucher** | **Geographic origin** | **identifier** |  |  |
| **1** | *Cinnamomum camphora* (L.) J. Presl | HITBC-BRG | SY32328 | XTBG, Yunnan, China | Yu Song |  |  |
| **2** | *Actinodaphne forrestii*(C.K.Allen) Kosterm. | HITBC-BRG | SY33187 | XTBG, Yunnan, China | Yu Song |  |  |
| **3** | *Litsea cubeba*(Lour.) Pers. | HITBC-BRG | SY34266 | XTBG, Yunnan, China | Yu Song |  |  |
| **4** | *Lindera communis*Hemsl. | HITBC-BRG | SY01432 | XTBG, Yunnan, China | Yu Song |  |  |
| **5** | *Persea americana* Mill. | HITBC-BRG | SY01359 | XTBG, Yunnan, China | Yu Song |  |  |
| **6** | *Machilns yunnanensis* Lecomte | HITBC-BRG | SY00401 | KIB, Yunnan, China | Yu Song |  |  |
| **7** | *Phoebe cabaleriei* (H. Lé v.) Y. Yang et Bing Liu | HITBC-BRG | SY34072 | KIB, Yunnan, China | Yu Song |  |  |
| **8** | *Neocinnamomum caudatum* (Nees) Merr. | HITBC-BRG | SY01561 | XTBG, Yunnan, China | Yu Song |  |  |
| **9** | *Caryodaphnopsis tonkinensis* (Lecomte) Airy Shaw. | HITBC-BRG | SY01520 | XTBG, Yunnan, China | Yu Song |  |  |
