# Supplementary material for: Transcriptome analysis reveals a composite molecular map linked to unique seed oil profile of Neocinnamomum caudatum (Nees) Merr
Source: BMC Plant Biol. 2018 Nov 26;18:303. doi: 10.1186/s12870-018-1525-9 (PMC6258453; doi:10.1186/s12870-018-1525-9)
Supplement: Supplementary file 13 — Gene-specific primer sequences for qRT-PCR validation. (DOCX 16 kb) [file 12870_2018_1525_MOESM13_ESM.docx]

**Table S2. Gene-speciﬁc primer sequences for qRT-PCR validation**

| **Gene** | **Unigene ID.** | **Forward primer (5'-3')** | **Reverse primer (5'-3')** |
| --- | --- | --- | --- |
| *KASI* | c86059_g1 | ATCCCTTACGCCATAACAAA | GACAAAGCTCTACATGCAACA |
| *KASII* | c89631_g1 | GAATATTGATCAACATGGCAG | TGATCTGCAAAATCCTGCTA |
| *SAD1* | c87644_g1 | CCTGAAGCCTGTTGAGAAGTGTTGG | CACCGTCAAGGGTGTTTAGCATTGT |
| *SAD2* | c79724_g1 | CATTTCCCGGCGACTTACACCA | TTGCTCCACCGGCTTCAGGA |
| *FATB* | c90063_g1 | AGAAACAGTGGACGAATCTTG | TCCGTACATGGTTAAGTGCA |
| *FATA* | *c89057*_g1 | ATGGATTTGCTACAACAACTACC | TTCATCATCACCCATTTGCT |
| *FAD2* | c87831_g1 | TTGATCACTTACCTGCAGC | GTAGTGGGGCATGTTAGAGAA |
| *FAD8* | c87670_g1 | ACTTGCATCATCATGACCATGAGCA | TGGTGAATCACATGAGTCCCAACAT |
| *ACT11* | c88651_g1 | TAATGGAACAGGAATGGTCA | CTCACAATACCATGCTCAATT |
