# Supplementary material for: Transcriptome analysis reveals a composite molecular map linked to unique seed oil profile of Neocinnamomum caudatum (Nees) Merr
Source: BMC Plant Biol. 2018 Nov 26;18:303. doi: 10.1186/s12870-018-1525-9 (PMC6258453; doi:10.1186/s12870-018-1525-9)
Supplement: Supplementary file 14 — The codon-optimized sequence of NcFATB. The full-length CDS of NcFATB was codon optimized and chemically synthesized in Genscript Co. Ltd. (China) for further validation of gene function in E. coli BL21 (DE3) cells. (DOCX 14 kb) [file 12870_2018_1525_MOESM14_ESM.docx]

>NcFATB_codon optimized

ATGGTGGCGACCGCGGCGGCGAGCGCGTTCTTTCCGGTTGCGAGCAGCAGCAGCAACGCGACCAGCGCGAAAGCGAGCATGATGCCGGACAACCTGGATGCGCGTGGCATTAAGCCGAAACCGGCGAGCAGCAGCGGTCTGCAGGTGAAGGCGAACGCGCACGCGAGCCCGAAGATCAACGGCACCAAAGTGAGCACCGAGAAGGGTGAAGACATGGTTACCAGCCACGCGGCGCCGCGTACCTTCATTAACCAGCTGCCGGATTGGAGCGTTCTGCTGGCGGCGATCACCACCATTTTTCTGGCGGCGGAGAAACAATGGACCAACCTGGACTGGAAGCCGCGTCGTCCGGACATGCTGGCGGATCCGTTCGGCATTGGTCGTTTTATGCAGGACGGCCTGATCTTCCGTCAAAACTTTGCGATCCGTAGCTACGAGATTGGTGCGGATCGTACCGCGAGCATCGAGACCCTGATGAACCACCTGCAGGAAACCGCGCTGAACCACGTGCGTAGCGCGGGTCTGCTGGGTGACGGTTTCGGTGCGACCCCGGAAATGAGCCGTCGTGATCTGATTTGGGTGGTTACCCGTATGCAAGTGCTGGTTGACCGTTATCCGGCGTGGGGCGATATCGTTGAGGTGGAAACCTGGGTGGGTGCGAGCGGCAAAAACGGTATGCGTCGTGACTGGCTGCTGCGTGATTGCCACACCGGCGAGATTCTGACCCGTGCGACCAGCGTGTGGGTTATGATGAACAAGAAAACCCGTCGTCTGAGCAAGATCCCGGACGAGGTTCGTGGCGAAATCGGTCCGTACTTTATTGAGAACGTGGCGATCGTTGAGGAAGACAGCCGTAAGCTGCAGAAACTGAACGAAAACACCGCGGATTACGTGCGTAAAGGCCTGACCCCGCGTTGGAGCGACCTGGATGTTAACCAACACGTGAACAACGTTAAGTATCTGGGTTGGATTCTGGAAAGCGCGCCGGTTAGCATCCTGGAGAGCCACGAACTGAGCTGCATGACCCTGGAGTATCGTCGTGAATGCGGCCGTGATAGCGTTCTGCAGAGCATGACCGCGCTGAGCGGTGGCGGTAGCGCGGTGGGCGGTAGCCCGGAGAGCGGTGTTGAGTGCGAACACCTGCTGCAACTGGAGAGCGGCAGCGAAGTGGTTCGTGGTCGTACCGAATGGCGTCCGAAAAGCGCGAACAACTTCGGTAGCATCATGGAGATTCCGGCGGAAAGCAGCTAA
